# Major Reorganization of Chromosome Conformation During Muscle Development in Pig

**DOI:** 10.3389/fgene.2021.748239

**Published:** 2021-10-05

**Authors:** Maria Marti-Marimon, Nathalie Vialaneix, Yvette Lahbib-Mansais, Matthias Zytnicki, Sylvie Camut, David Robelin, Martine Yerle-Bouissou, Sylvain Foissac

**Affiliations:** ^1^ GenPhySE, Université de Toulouse, INRAE, ENVT, Castanet Tolosan, France; ^2^ Université de Toulouse, INRAE, UR MIAT, Castanet-Tolosan, France

**Keywords:** Hi-C, chromosome conformation, chromatin structure, telomeres, pig, development, fetal muscle, 3D genome architecture

## Abstract

The spatial organization of the genome in the nucleus plays a crucial role in eukaryotic cell functions, yet little is known about chromatin structure variations during late fetal development in mammals. We performed *in situ* high-throughput chromosome conformation capture (Hi-C) sequencing of DNA from muscle samples of pig fetuses at two late stages of gestation. Comparative analysis of the resulting Hi-C interaction matrices between both groups showed widespread differences of different types. First, we discovered a complex landscape of stable and group-specific Topologically Associating Domains (TADs). Investigating the nuclear partition of the chromatin into transcriptionally active and inactive compartments, we observed a genome-wide fragmentation of these compartments between 90 and 110 days of gestation. Also, we identified and characterized the distribution of differential *cis*- and *trans*-pairwise interactions. In particular, *trans*-interactions at chromosome extremities revealed a mechanism of telomere clustering further confirmed by 3D Fluorescence *in situ* Hybridization (FISH). Altogether, we report major variations of the three-dimensional genome conformation during muscle development in pig, involving several levels of chromatin remodeling and structural regulation.

## 1 Introduction

Deciphering the mechanisms that govern gene expression regulation is essential for understanding the fundamental biological changes occurring under different physiological conditions. In this context, genome organization has been proven to be a major player in the regulation of gene expression (Bonora et al., 2014; Bonev and Cavalli, 2016; Long et al., 2016). Understanding the relationship between genome organization and gene expression needs a deep knowledge of chromatin structure and folding, which has been made possible by the development of three-dimensional (3D) techniques like 3D DNA Fluorescence *in situ* Hybridization (FISH) and Chromosome Conformation Capture assays (Dekker et al., 2002; Davies et al., 2017), including its genome-wide version Hi-C (Lieberman-Aiden et al., 2009). By identifying pairs of genomic regions in direct physical contact or in close spatial proximity within the nucleus, hereafter referred as “interactions”, these approaches revealed several features of the genome architecture. For instance, individual chromosomes occupy discrete territories in the interphase nuclei, the so-called chromosome territories (Cremer and Cremer, 2001; Bolzer et al., 2005; Cremer et al., 2006), which may intermingle at the interface regions allowing *trans*-chromosomal interactions (Branco and Pombo, 2006; Nagano et al., 2013). Moreover, chromosomes have been found to be organized in two main types of large regions with different features in terms of genome topology, chromatin state and gene expression. These regions of several megabases are the A and B compartments that correspond respectively to open transcriptionally active and close inactive chromatin. While A compartments are associated with euchromatic, gene-rich and DNase I hypersensitive regions, B compartments are considered as transcriptionally inert, heterochromatic, nuclear lamina-associated, gene-poor and DNase I insensitive (Lieberman-Aiden et al., 2009; Gibcus and Dekker, 2013; Bonora et al., 2014). Although these compartments could be further segmented considering finer epigenetics features (Rao et al., 2014) or associated with exceptional euchromatin/heterochromatin organisations (Feodorova et al., 2020), we will simply refer to the general A/B definition hereinafter. At a smaller scale, genomic regions of about 1 Mb with a high density of *cis*-interactions, named Topologically Associating Domains (TADs) (Dixon et al., 2012; Nora et al., 2012; Sexton et al., 2012) have been shown to play a role in regulating gene expression during key biological processes like development (Gibcus and Dekker, 2013; Lupiáñez et al., 2015).

To gain insight into the establishment, the dynamics and the function of these genomic structures, several studies have characterized them in various cell types and compared them within or, sometimes, between species (Dixon et al., 2012; Rudan et al., 2015; Foissac et al., 2019). Various comparisons have been performed during early embryo development (Zheng and Xie, 2019), between different cell lines (e.g., embryonic and mesenchymal stem cells Dixon et al. (2015)), from distinct differentiation states (e.g., during neural differentiation Bonev et al. (2017), or during B cell fate commitment Boya et al. (2017); Lin et al. (2012)). Such comparisons efficiently revealed strong differences between distinct cell types, requiring few biological replicates (often simple duplicates), but they did not provide information about the heterogeneity and the dynamics of the genome 3D structure for a specific cell type. The development of single-cell Hi-C (Nagano et al., 2013) made possible to determine whole genome structures in single haploid (Stevens et al., 2017) or diploid cells (Tan et al., 2018). Recent applications of single-cell Hi-C revealed various degrees of heterogeneity in genome 3D conformation among several cell lines (Ramani et al., 2017; Finn et al., 2019).

Despite all these efforts, little is known about the status and the dynamics of chromosome organization in animal cells from most of the organized tissue types, with notable exceptions like brain and liver for instance (Vietri Rudan et al., 2015; Won et al., 2016; Harewood et al., 2017; Foissac et al., 2019). Regarding skeletal muscle, Hi-C experiments have been performed on cultured cells (Doynova et al., 2017; He et al., 2018) and on adult muscle (Schmitt et al., 2016), but little is known about chromosome organization in this type of differentiated cells during late development. To assess whether significant structural dynamic modifications could also be detected there, we characterized the 3D genome organization of porcine *longissimus dorsi* muscle cells during late fetal development (days 90 and 110 of gestation) by adapting the *in situ* Hi-C protocol (Rao et al., 2014) to fetal frozen tissues. This period, which covers almost the entire last month of gestation, is known to be crucial for porcine muscle development and maturity, involving major reorganizations of the transcriptomic and proteomic programs (Voillet et al., 2014, 2018). By performing the experimental assays on tissue samples from different fetuses (three replicates per group of the Large White breed), we characterized the genomic structure of pig muscle cells at various levels of organization, providing high-resolution Hi-C interaction maps, TAD and A/B compartment annotations. Comparing samples from 90 vs 110 days of gestation allowed the identification of major topological differences between the two groups, in line with previous results from transcriptome characterization. In addition, these results completed and further expanded previous studies which identified *trans* interactions involving genes that are key players for fetal muscle growth and development (Lahbib-Mansais et al., 2016; Marti-Marimon et al., 2018). Overall, this study sheds a new light on the description of dynamic changes of the 3D genome occurring during transcriptional switches in the expression programs of differentiated cells.

## 2 Materials and Methods

Experimental and computational resources used in this study are listed in Supplementary Table 3, including names of chemical reagents, kits and software versions.

### 2.1 Animals and Samples

For Hi-C and FISH experiments, *longissimus dorsi* fetal porcine muscle samples were collected from the European Large White (LW) breed (F1 LW × LW). Specifically, three 90 days gestation male littermates and three 110 days gestation (two male littermates and one female) were used for Hi-C assays. For FISH experiments, muscle samples were collected from different fetuses (one at 90 days gestation and one at 110 days) of those in which Hi-C experiments were performed. All the fetuses used in this study were obtained by caesarean after euthanasia of healthy wild type sows and fetuses. No special breeding conditions (feeding, housing, treatment) were applied.

The experimental design was approved and authorized by the ethical committee (#84) in animal experimentation of the French Ministry of National Education, Higher Education, and Scientific Research (authorization #02015021016014354). The experiment authorization number for the experimental farm GenESI (Genetics, testing and innovative systems experimental unit) is A17661. The procedures performed in this study and the treatment of animals complied with European Union legislation (Directive 2010/63/EU) and French legislation in the Midi-Pyrénées Region of France (Decree 2001–464). All the details about the animals and samples have been registered in the BioSamples public repository (https://www.ebi.ac.uk/biosamples) in agreement with the FAANG best practices guidelines (https://www.faang.org/data-share-principle) and are available using the accession SAMEA7390788.

### 2.2 3D DNA FISH Experiments

#### 2.2.1 Cells and Probes Preparation

Fetal muscle tissue was obtained from the *Longuissimus dorsi* muscle of 90- and 110-days of gestation Large White (LW) pig and prepared as described in Lahbib-Mansais et al. (2016); Marti-Marimon et al. (2018). Stored muscle fibre packets were permeabilised for 5–8 min in cytoskeleton extraction buffer (100 mM NaCl, 300 mM sucrose, 3 mM MgCl2, 10 mM PIPES pH 6.8) containing 0.5% Triton X 100 and then fixed in cold 4% paraformaldehyde for 5 min. After washing in cold PBS, muscle packets were manually dilacerated directly on Superfrost glass slides (CML, Nemours, France) to isolate individual fibres, and air-dried before adding DNA probes for *in situ* hybridization. Bacterial artificial clones (BACs) containing specific subtelomeric sequences of porcine chromosomes 2, 9, 13 and 15 were chosen as selected by Mompart et al. (2013): SSC2p (PigI-370D12), SSC9q (PigI-441D12, PigI-564B6), SSC13q (PigI-39F7) and SSC15q (PigI-899B10). These BACs were isolated from a porcine BAC library (CRB-Anim, INRA, 2018. Biological Resource Centres for domestic animals of AgroBRC, doi: http://doi.org/10.15454/1.5613785622827378E12). For multiple-label experiments, approximately 120 ng of each BAC DNA was random-priming labelled directly by incorporation of dUTP Alexa Fluor (488 or 568) or indirectly with Biotin-6-dUTP detected by immuno-FISH (Bioprime DNA labelling kit, Invitrogen, Cergy Pontoise, France). Three combinations of p or q telomeres probes of different pairs of chromosomes: (SSC2qter – SSC9qter), (SSC13qter – SSC9qter) and (SSC15qter – SSC9qter) were chosen to test their rate of association as suggested by Hi-C.

#### 2.2.2 3D DNA Fluorescence in Situ Hybridization

3D DNA FISH experiments were conducted as described in Lahbib-Mansais et al. (2016) with slight modifications. Probes were resuspended in hybridization buffer (50% formamide, 10% dextran sulphate, 2 mg/ml BSA, 2X SSC) at a final concentration of 110 ng/μL. Nuclear DNA of fibers and probes were simultaneously heat-denatured at 74°C for 7 min on the slide and then incubated overnight at 37 C in a DAKO hybridizer. Post-hybridization washes were then performed with gentle agitation, first twice in 2X SSC at 40°C for 6 min, then in 2X SSC, 50% formamide pH 7.0 at 40°C for 6 min, and finally twice for 10 min in 2X SSC, then in PBS at RT. When a biotin labelled probe was used, biotins were detected with streptavidin Alexa 568 or 488 at a final concentration of 5 μg/ml for 1 h at RT.

3D acquisitions were performed at the T.R.I. Genotoul (Toulouse Réseau Imagerie, http://trigenotoul.com/en) imaging core facility in Toulouse (France). Image stacks were collected using a Leica SP8 confocal microscope (Leica Instruments, Heidelberg, Germany) equipped with an oil immersion objective (plan achromatic 63 × N.A. = 1.4). The Z-stacks (around 80 confocal planes per capture) were acquired at 1,024 × 1,024 pixels per frame using an 8-bit pixel depth for each channel at a constant voxel size of 0.06 × 0.06 × 0.3 μm.

### 2.2.3 Telomere Association Analysis

Images were analyzed with specific software NEMO (Iannuccelli et al., 2010), distributed under the creative commons license that can be freely downloaded from https://forge-dga.jouy.inra.fr/projects/nemo. Segmentations and 3D measurements between signals (center-to-center distance) were done as described in Lahbib-Mansais et al. (2016). Euclidean distances were computed with respect to the *x*, *y* and *z* resolutions. Given the resolution on the *z* axis, at least three pixels corresponding to 0.9 μm (0.3 × 3) were required for a high resolution between two separate signals; consequently, 1 μm was chosen as the upper cut-off for associated signals. For each combination of telomeres, nuclei were only analyzed when four signals (corresponding to the chosen telomeres of two chromosomes) were present. “Associated” signals were considered when they are separated by a distance (d) ≤ 1 μm, as done in Lahbib-Mansais et al. (2016). We determined for each combination of telomeric pairs how many nuclei were found associated among about 100 observed nuclei.

Significance of the difference in association between d90 and d110 was assessed using a χ^2^ test to compare generalized linear models of the binomial family with a fixed telomeric pair covariate and including, or not, the condition as a second covariate (see Supplementary Methods).

### 2.3 Hi-C Experiments

#### 2.3.1 Hi-C Protocol

Hi-C experiments were performed as previously documented (Foissac et al., 2019), with slight modifications to adapt the Hi-C experiments and libraries to fetal muscle tissues (see Supplementary Methods).

#### 2.3.2 Hi-C Quality Controls

After DNA digestion with HindIII, and filling-ligation of the digested ends, the HindIII target site disappears and a NheI restriction site is created instead. To check the efficiency of the Hi-C assays, PCR were performed around one HindIII restriction site with two forward primers (Fwd1: 5′ TCT​GGG​CAG​GTC​ACT​CAT​T 3′; Fwd2: 5′ TCT​CGG​GAT​GCT​GAG​TGT​TT 3′; product size = 425 bp). A reverse primer combined with Fwd1 was used as a control (Rv1: 5′ AAA​CAC​TCA​GCA​TCC​CGA​GA 3′; product size = 465 bp). In Hi-C, some religation events allow switching the sense of one DNA fragment and PCR amplification with the two forward primers. The PCR amplification products from the couple of forward primers were digested either with HindIII or NheI (product sizes = 201 + 215 bp). In control tubes (no filling of digested ends), HindIII should cleave the PCR products while NheI should not. In Hi-C tubes, NheI should cleave most of the PCR products while HindIII should cleave only a small fraction.

#### 2.3.3 Hi-C Library Production

1.4 μg of DNA from the Hi-C experiments were fragmented with a Covaris machine. Then, 0.55 volumes of CleanPCR magnetic beads were added to the fragmented DNA to select fragments <600 bp (5 min incubation and keeping the supernatant), and 0.7 volumes of beads were added again (5 min incubation and removing supernatant) to remove fragments <200 bp. Then beads were washed with 80% ethanol and DNA was recovered with Resuspension Buffer. To purify biotinylated DNA, one volume of M-280 streptavidin magnetic Dynabeads was added and after 15 min incubation, the supernatant was removed and the beads were washed 4 times with beads wash buffer (Nextera Mate Pair Preparation Kit, Illumina) and twice with Resuspension buffer. From this point, all steps were performed while DNA remained attached to the beads. To repair DNA breaks, 60 μL of water and 40 μL of End Repair Mix 2 (TruSeqNano DNA library prep, Illumina) were added and incubated 30 min at 30°C, and then beads were washed as explained before. To allow the adapters ligation, an “A” nucleotide was added to the 3’ ends by adding 17.5 μL of water and 12.5 μL of A-Tailing Mix (TruSeqNano DNA library prep, Illumina) and incubating 30 min at 37°C and then 5 min at 70°C to inactivate the enzyme. To ligate the adapters to the DNA extremities, 2.5 μL of Resuspension Buffer, 2.5 μL of DNA Ligase Mix and 2.5 μL of DNA Adapter Index (TruSeqNano DNA library prep, Illumina) were added (10 min incubation at 30°C, then 5 μL of Stop ligation Buffer) and then beads were washed as before. DNA was amplified by 12 PCR cycles (15 s at 98°C–30 s at 60°C–30 s at 72°C) by resuspending beads in 50 μL of PCR mix (25 μL Enhanced PCR mix, 5 μL PCR primer Cocktail and 20 μL water, TruSeqNano DNA library prep, Illumina). To recover DNA from the beads, 0.6 volumes of CleanPCR magnetic beads were added and incubated 5 min, and then washed twice with 80% ethanol, resuspended in 30 μL of Resupension Buffer and after placing in a magnetic rack, supernatant containing the libraries was recovered. Libraries size was controlled with the Fragment Analyzer (FA) and quantified by qPCR. In addition, an aliquot was digested by using the NheI and HindIII enzymes to verify if selected fragments are the ones containing the filled-in biotinylated religation sites as done in Belton et al. (2012). Libraries were sequenced in pool in one HiSeq3000 lane to validate their quality. For depth sequencing, the pool was paired end (PE) sequenced in 11 lines of a HiSeq3000 (reads size = 150 bases), producing from ∼ 476–685 M read pairs per library in total (see Supplementary Table 2).

### 2.4 Hi-C Data Analysis

#### 2.4.1 Hi-C Reads and Interaction Matrices

The 3,447,428,742 Paired-End reads were processed using HiC-Pro v2.9.0 (Servant et al., 2015) as previously reported (Foissac et al., 2019). The bioinformatics analysis includes the following steps (see Supplementary Methods for more details).

• Read mapping was performed on the Sscrofa11.1 genome assembly version using Bowtie two v2.3.3.1 (Langmead and Salzberg, 2012).
• Interaction matrices were generated from valid pairs at various resolutions depending on the bin size. Most of the subsequent analyses were performed at the 500 Kb resolution apart from few exceptions (TAD detection for instance was performed at the 50 Kb resolution). A total of six interaction matrices were obtained per resolution (n = 3 (replicates) × 2 (groups)). Additionally, merged interaction matrices were computed by summing the interaction values of the three matrices for each group. Considering the high number of unassembled scaffolds in the pig genome Sscrofa11.1 version and given the fact that samples from both genders were collected, we focused our analysis on the 18 assembled autosomes to avoid potential effects of the sexual chromosomes on the results.
• Interaction matrices were displayed using Juicebox (Durand et al., 2016) and HiTC R/Bioconductor package v1.18.1 (Servant et al., 2012).
• Interaction matrices were normalized per chromosome using the non-parametric iterative correction and eigenvector decomposition (ICE) method when needed (Imakaev et al., 2012).
• Replicability between interaction matrices was assessed using the replicability index of Yang et al. (2017) as implemented in the R/Bioconductor package hicrep.
• Maximal resolution was computed following Rao et al. (2014): a given resolution (bin size) can be claimed if, at that resolution, 80% of the bins or more contain at least 1,000 interactions. The proportion of bins with a cumulated number of valid interactions higher than 1,000 was therefore computed for different resolutions (from 100 to 5 Kb) for each individual (sample) and for the merged (group) matrix.

### 2.4.2 TADs Calling and Comparison

TADs were predicted per chromosome from raw interaction matrices [n = 3 (replicates) × 2 (groups) × 18 (autosomes)] at 50 Kb resolution with the Arrowhead method of the Juicer tool v1.5.3, using the -k KR parameter to ensure matrix balancing normalization. TAD finding was performed on individual matrices of each replicate separately (to assess group replicability) and on the merged matrices [n = 2 (groups) × 18 (autosomes)] to obtain a set of TADs for each group (90/110 days of gestation). To identify TADs that are consistently predicted from different replicates and group-specific TADs, we performed pairwise comparisons of TAD sets from different replicates using bedtools (v2.26.0). A mutual overlap of 90% similarity was required with the parameters -f 0.9 -r.

Insulation capacity of TAD boundaries was computed as previously described (Foissac et al., 2019) using the local interaction score. In brief, considering all valid interactions around the same TAD boundary (*i.e.*, both reads being not further than 500 Kb from the boundary) the interaction score corresponds to the proportion of valid interactions across the boundary. IS scores were normalized by cyclic loess (Ballman et al., 2004) using csaw (Lun and Smyth, 2015) (see Supplementary Methods for more details).

### 2.4.3 CTCF Prediction

The position specific frequency matrix corresponding to the CTCF-binding motif was recovered from the JASPAR Transcription Factor Binding Sites (TFBS) catalogue [http://jaspar.genereg.net, Mathelier et al. (2016)]. CTCF genomic occurrences were predicted by running FIMO v.4.11.1 Grant et al. (2011) with the JASPAR CTCF frequency matrix on the Sscrofa11.1 genome. Then, the average density of CTCF predicted motifs with respect to TAD positions was obtained using bedtools v2.26.0 map and coverage functions Quinlan (2014).

### 2.4.4 A/B Compartments Detection

A and B compartments were obtained using the PCA approach described in Lieberman-Aiden et al. (2009), as implemented in the R/Bioconductor package HiTC (Servant et al., 2012). A/B compartment identification was performed on intra-chromosome interaction matrices at 500 Kb resolution on individual interaction matrices [n = 3 (replicates) × 2 (groups) × 18 (autosomes)] and on the merged interaction matrix (n = 18 autosomes). Boundaries between A and B compartments were identified according to the sign of the first PC (eigenvector). Bins that were not assigned to any compartment due to a lack of data in some samples were not considered in subsequent integrative analyses. As an additional control, A/B compartments were also obtained by using the eigenvalue method of the Juicer tool (Durand et al., 2016), which led to similar results.

The difference between the number of compartments in the two groups was assessed with a Poisson GLM: log (*y*_*ijk*_) ∼ *α c*_*ijk*_ + *β*_*k*_, with *y*_*ijk*_ the number of compartments in chromosome *j* from sample *i* in group *k*, *c*_*ijk*_ the total number of valid interactions in chromosome *j* from sample *i* in group *k*, *α* its estimated effect on the number of compartments, and *β*_*k*_ the estimated effect of the group on the number of compartments, which was tested for being significantly different from 0 [test with n = 2 (groups) × 3 (samples) × 18 (chromosomes) observations].

### 2.4.5 Detection of Differential Interactions

A differential analysis was performed to extract interactions that were significantly differentially connected between the two groups (90 and 110 days of gestation). This analysis was performed on raw count data from the 18 autosomes at the 500 Kb resolution (the differential analysis was thus performed with two groups and *n* = 3 replicates in each group). A method similar to the one described in Lun and Smyth (2015), with some adaptations, was used to perform this task. In brief (see Supplementary Methods for more details):

• Low count interactions with less 30 reads across the six samples (5 reads per sample on average) were discarded from the analysis.
• Interaction values were normalized using a non-linear normalization method Ballman et al. (2004) based on a fast cyclic loess algorithm implemented in the R/Bioconductor package csaw (Lun and Smyth, 2016).
• Differential analysis was performed using a Generalized Linear Model (GLM) based on the Negative Binomial (NB) distribution with a group fixed effect (two-level factor: 90/110 days). The model was estimated with the implementation of the R/Bioconductor package edgeR (Robinson et al., 2010; McCarthy et al., 2012) and log ratio tests were used to assess the significativity of the group effect on each bin pair interaction. *p*-values were genome wide corrected using (Benjamini and Hochberg, 1995) procedure to control the False Discovery Rate (FDR).

### 2.4.6 Characterization of BODIs

As a single genomic bin can be involved in multiple Differential Interactions (DI) genome-wide with various logFC values, we looked for bins with a large prevalence of interactions of the same logFC sign, either mostly positive or mostly negative. A minimum ratio of 90% of DI with the same sign was required to identify “positive” or “negative” bins, possibly indicating regions that undergo a chromatin contraction or opening, respectively. Bins with a mixture of positive and negative DI were considered as undefined. Adjacent bins with the same sign (either positive, negative, or undefined) were merged into Blocks Of Differential Interactions (BODIs). This analysis was performed considering only intra-chromosomal DIs (in *cis*).

To assess the existence of an enrichment of large positive and negative BODIs given the relative proportions of positive and negative individual DIs, a permutation test was performed: at each permutation, logFC values were shuffled genome-wide across DIs. The same 10:1 threshold was applied to define prevalently positive and negative bins and adjacent bins of the same type were merged to identify “expected BODIs” under the null hypothesis (no specific trend of positive/negative bins to cluster consecutively). The resulting size distributions of positive, negative and undefined BODIs were compared with that of observed BODIs, and the *p*-value was computed, as the number of times expected BODIs were at least as frequent as the observed ones across 100 permutations for a given size and type.

The comparison of BODIs with A/B compartments was done by computing the proportion of the positive, negative and undefined BODIs that overlapped A or B compartments in terms of genomic space. The resulting block composition was therefore obtained using the bedtools coverage function on BODIs of each size and compartments of each type. As most of the compartmentalization is stable across samples, the A/B compartments obtained on the merged general matrix was used. Since A and B compartments cover roughly the same genomic space in total, no large difference should be observed between the A and B composition of positive and negative BODIs. Significance was assessed using Fisher’s exact test between the compartment type (A/B) and the BODI types (positive/negative).

### 2.5 Gene Expression Integrative Analysis

#### 2.5.1 Expression Data

Expression data were obtained from a previous transcriptome study of skeletal muscle in pig during development using microarrays (Voillet et al., 2014). The dataset consists of 44,368 probe measurements for 17 samples (LW animals) at two different gestational stages: eight samples at 90 days and nine samples at 110 days. A precise description of the experimental design and data collection can be found in Voillet et al. (2014). Normalized expression data (log_2_ transformed) and sample information are available in NCBI (GEO accession number GSE56301). Log_2_ transformed expressions and log fold change (logFC) of these expression values at 90 vs 110 days (reference time point: 90 days) were used in our integrative analyses. Since the microarray was originally designed on a former version of the pig genome, probes were remapped on the Sscrofa11.1 assembly version and further filtered (see Supplementary Methods for more details).

#### 2.5.2 Density and Expression Level of Genes in A/B Compartments

To compare the gene density in A vs B compartments, a gene density value was first computed for each compartment by dividing the number of distinct gene IDs included in the compartment (using bedtools map) by the size of the compartment. Resulting gene density distributions were then compared between A and B compartments. Normality of the gene density was tested using Shapiro-Wilk normality test and rejected for all types of compartments in both groups (*p*-values < 2.2*e* ^−16^ overall, for n = 349 and 322 A and B compartments respectively). Wilcoxon tests were then used to assess the significance of the difference in gene density in A vs B compartments.

To compare the average gene expression in A vs B compartment, we computed for each compartment the mean expression value of its genes using bedtools map separately for the two gestational ages. Normality of the average gene expression was tested using Shapiro-Wilk normality tests and rejected for both A and B compartments (*p*-values = 2.58*e* ^−5^ and 1.08*e* ^−3^ for n = 344 and 292 A and B compartments with at least one expressed gene, respectively). Wilcoxon tests were then used to assess the significance of the difference in gene expression in A vs B compartments.

To investigate the dynamic of expression in compartment-switching regions, we considered the logFC expression values of the genes and split them into compartment-switching categories using bedtools: no switch, A to B, B to A. Normality of the logFC expression values was tested using Shapiro-Wilk normality tests for genes in all types of compartments except for compartments with no switch (n = 7, 511 genes in these compartments, above the applicability condition of the test) and rejected for both types of compartments (*p*-values = 1.2*e* ^−3^ and 4.6*e* ^−6^, for *n* = 60 and 174 genes in compartments switching from A to B and from B to A, respectively). Wilcoxon tests were then used to assess the significance of the difference in logFC expression values in each compartment type.

## 3 Results

### 3.1 Genome-wide Maps of Chromosomal Interactions in Fetal Porcine Muscle Tissue

**FIGURE 1:**
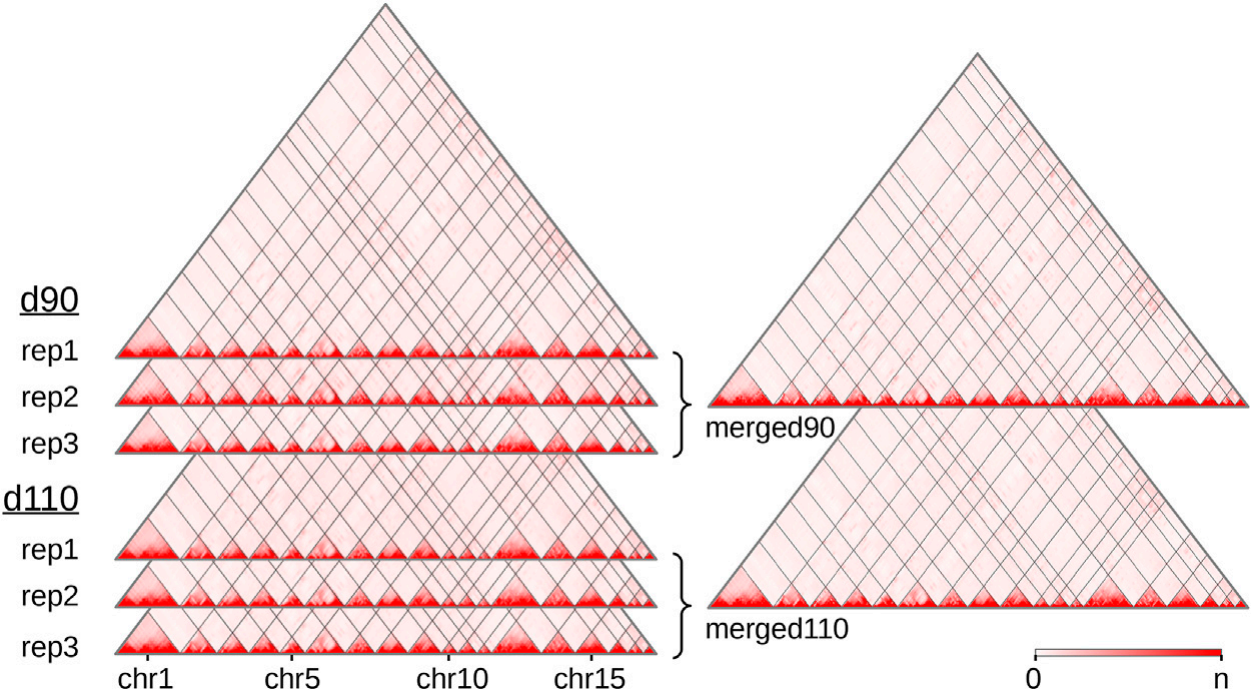
Hi-C interaction maps of the porcine genome in fetal muscle. Interaction matrices of three biological replicates from two experimental groups (90 and 110 days of gestation) were displayed with the Juicebox tool, before and after merging them by group. The color intensity indicates the number of interactions between pairs of genomic loci (*x*-axis, 500 Kb per bin). Since the color scale is generated for each matrix independently, the highest intensity corresponds to the following values of *n*: 16,103 (rep1), 13,257 (rep2) and 11,461 (rep3) for d90, 13,022 (rep1), 7,150 (rep2) and 16,070 (rep3) for d110, 43,029 for merged90 and 37,866 for merged110. As the *Sus scrofa* v11.1 assembly version contains 613 scaffolds, only the 18 assembled autosomes are displayed. See also Supplementary Table 2.

We produced and sequenced Hi-C libraries from muscle samples of six pig fetuses (Supplementary Table 1): three replicates at 90 days of gestation (“d90” group) and three replicates at 110 days of gestation (“d110” group). We obtained ∼7 billion reads in total across the six samples. After trimming the sequences when needed, we could map from 63 to 73% of the pairs on the *Sus scrofa* v11.1 reference genome (Supplementary Table 2). These proportions are lower than usually reported with human or mouse cells (Rao et al., 2014). This could be explained by several reasons, including the slightly lower quality of the porcine genomic sequence compared with the human or murine ones, and the nature of the biological material used here (frozen samples of fetal muscle). In each library, nevertheless, most of the mapped pairs showed consistent mapping configurations with respect to the genomic positions of the HindIII restriction sites (Yaffe and Tanay, 2011). Those were labeled as “valid interactions” (Supplementary Table 2). Overall, we obtained between 112 and 260 M valid interactions per sample from which we generated six individual interaction matrices, one per sample (Figure 1). To precisely assess the general similarity between matrices, we computed the replicability index (Yang et al., 2017) between all pairs of matrices from different groups (*i.e.*, d90 vs d110) and from the same group (see *Hi-C reads and interaction matrices* and Supplementary Methods). By considering matrices from a previous study made on liver samples in adult pigs (Foissac et al., 2019), we could also compute the similarity measure between matrices from different tissues and development stages. As expected, the highest replicability index was obtained between replicates from the same group (0.92 on average, compared to 0.87 between groups and 0.67 between tissues). Adding counts from matrices of the same group generated two high-density matrices named “merged90” and “merged110” (Figure 1). More precisely, maximum matrix resolutions as defined by Rao et al. (2014) were 25 Kb on average per individual sample, 15 Kb for the merged110 matrix and 10 Kb for the merged90 matrix (see Methods).

### 3.2 A Complex Landscape of Stable and Group-specific TADs

**FIGURE 2:**
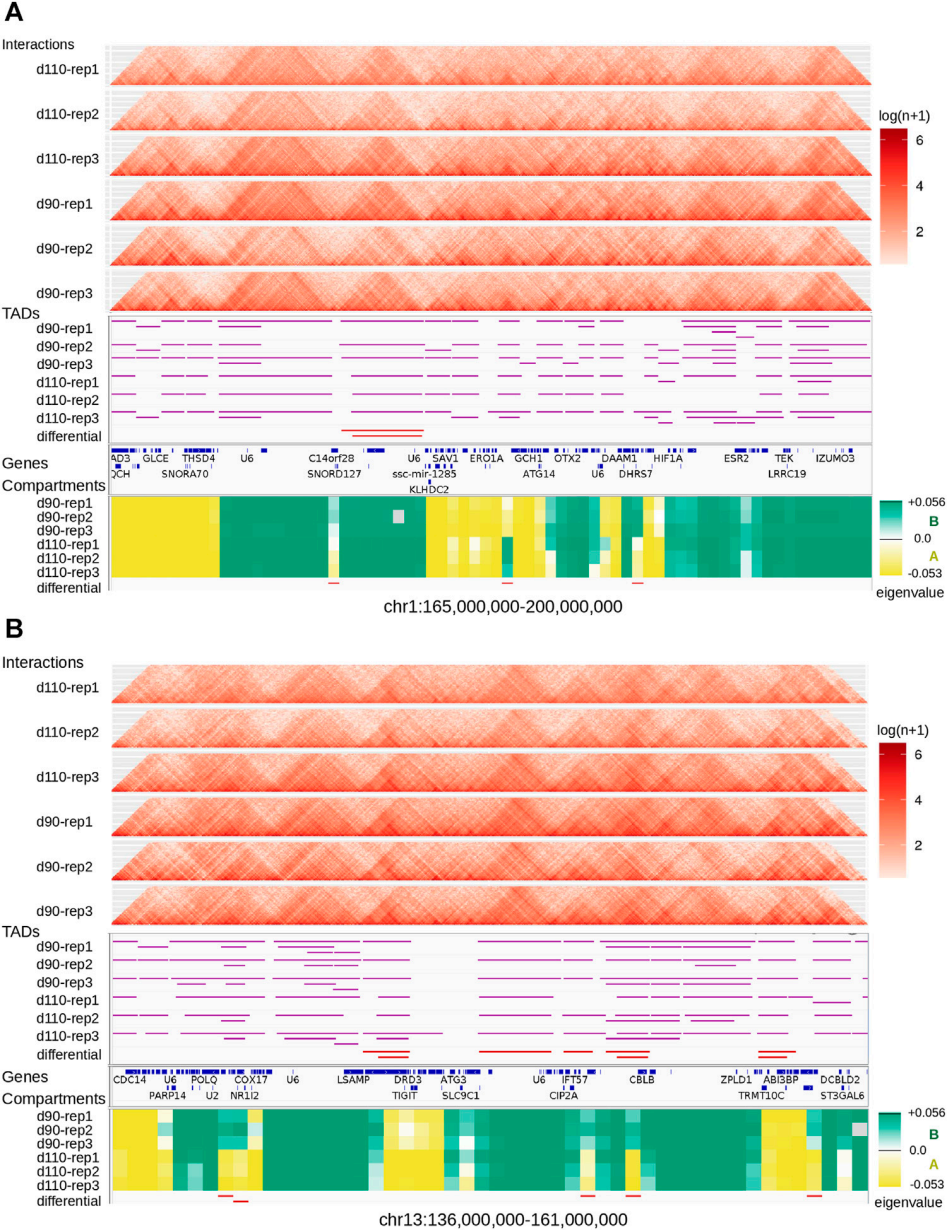
Landscape of topological features in the pig genome. Hi-C interaction maps **(top, heatmaps)**, TADs **(middle, horizontal purple lines)**, and genomic compartments **(bottom, green/yellow eigenvalues for A/B compartments respectively)** are displayed for the six samples at two loci of the pig genome: one on chromosome 1 **(A)** and one on chromosome 13 **(B)**. Annotated genes are listed between TADs and compartments. The last track (at the bottom) shows regions with a consistent switch of compartment for all replicates (AAA → BBB or BBB → AAA).

**FIGURE 3:**
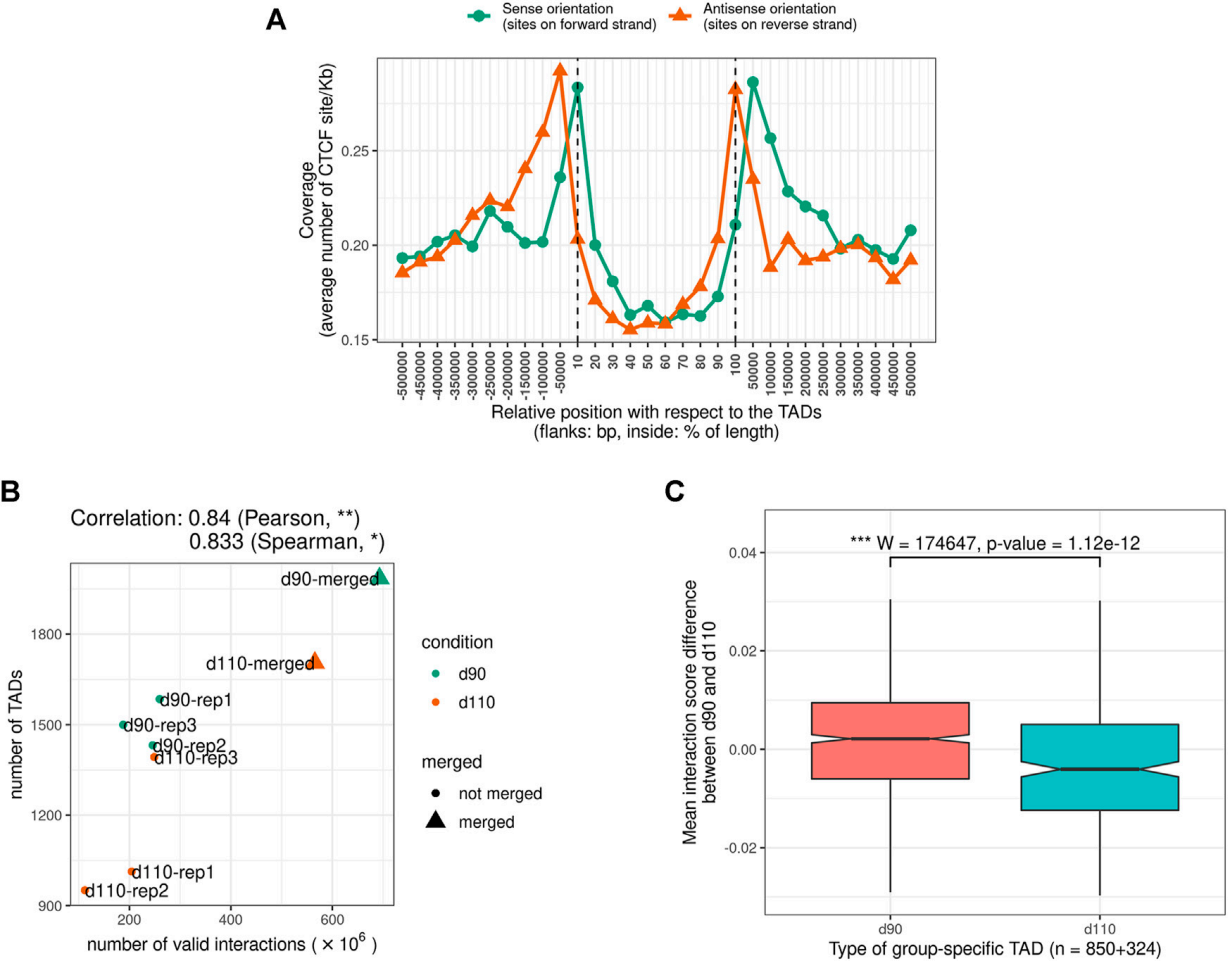
Characteristics of Topologically Associating Domains (TADs) **(A)** The genomic distribution of CTCF binding sites over TAD regions shows an accumulation of sites at the TAD boundaries in the expected inwards orientation, meaning forward and reverse sites respectively at the beginning and at the end of TADs. Flanking TADs explain the shifted peaks corresponding to sites in the outwards orientation **(B)** Correlation between Hi-C matrix density (number of interactions) and number of predicted TADs **(C)** Distributions of Interaction Score Differences between 90 and 110 days of gestation for boundaries of d90-and d110-specific TADs.

We looked for Topologically Associating Domains (TADs) in each interaction matrix (see *TADs calling and comparison*) and identified 1,312 TADs per sample on average, with 84.7% of the genome being part of a TAD in at least one of the samples. Examples are displayed in Figure 2. The median TAD size of 1,200 Kb (Supplementary Table 2) was consistent with previous results in human and mouse (Dixon et al., 2012; Zufferey et al., 2018). In addition, computationally-predicted CTCF binding sites accumulated at TAD extremities in the expected orientation (Figure 3A, Rao et al. (2014)).

The number of TADs differed between samples (from 951 to 1,585 per sample and up to 1,985 in the merged90 matrix, Supplementary Table 2). Part of this variability could be explained by the difference in the number of interactions per matrix. Indeed, computational TAD detection is known to be sensitive to variations in matrix density that can result from differences in sequencing data quantity or library complexity for instance Dali and Blanchette (2017); Zufferey et al. (2018). Consistently, we observed a significant correlation between the number of valid interactions and the number of TADs across samples (Pearson correlation coefficient = 0.83, *p*-value = 9.10*e* ^−3^, Figure 3B). The position of the TADs also differed between samples, although the global structure appeared stable overall (Figure 2). TAD conservation across cell types and model species has been widely reported and discussed with various degrees of circumspection (Barutcu et al., 2015; Cubeñas Potts and Corces, 2015; Dixon et al., 2015; Fraser et al., 2015; Doynova et al., 2017; Eres and Gilad, 2020; Sauerwald et al., 2020). Here, we sought to investigate TAD stability within the same tissue, by comparing samples from either the same or different groups (d90 vs d110). We considered that two TADs were identical when they overlapped with each other by 90% of their length. Pairwise comparisons of samples from the same group resulted in 1,785 identical TADs out of 2,625 on average (68.0%). As expected, this proportion was lower when comparing samples from different groups, with 1,457 identical TADs out of 2,625 on average (55.5%). Nevertheless, the observation that most of the TADs are shared within any pair of samples seemed to confirm a general stability of the TAD structure. This stability decreased drastically when requiring identity across more than two samples: for instance, only 29.0% of the TADs (2,286 out of 7,874) were identical across all the six samples. Even accounting for the presence of samples from different groups, this observed variability within the same tissue illustrates the issue of estimating TAD stability using a limited number of replicates (Sauerwald et al., 2020). The set of identical TADs in all six samples is provided in Supplementary File 1.

The difference between the proportions of identical TADs in samples from the same vs from different groups prompted us to investigate the existence of “group-specific” TADs. To find them, we considered all TADs with an identical TAD in each of the three replicates within the same group but no identical TAD in any replicate from the other group. This simple filtering process led us to a small set of 252 distinct group-specific TADs (201 for d90 and 51 for d110). It should be noted that visual inspection of the interaction matrices at the corresponding genomic positions did not show striking differences in the TAD patterns between groups (Figure 2). In order to confirm the consistency between the group-specific TADs and the raw matrix data, we computed and compared the local Interaction Score of the group-specific TAD boundaries in both groups. The Interaction Score (IS) is defined as the proportion of interactions across the midpoint of a given genomic region (see *TADs calling and comparison*) and can be used to assess the insulation property of TAD boundaries (Foissac et al., 2019). We computed the IS at each TAD boundary for each sample and computed the difference of the mean score between the d90 and the d110 groups (hereafter referred to as “ΔIS”). Negative ΔIS indicates a relative loss of interactions between 90 and 110 days. They should therefore characterize TAD boundaries that became stronger or that appeared during gestation, as one would expect for d110-specific TADs. Symmetrically, positive ΔIS indicates a gain of interactions and should therefore characterize TAD boundaries that became more permissive or disappeared during gestation. As expected, comparing the ΔIS values of the d90-and d110-specific TAD boundaries showed that the average ΔIS was positive for boundaries of d90-specific TADs but negative for boundaries of d110-specific TADs (Figure 3C). Moreover, the difference was statistically significant (*p*-value < 2*e* ^−7^, Wilcoxon test), supporting that group-specific TADs exhibit opposite dynamics of boundary strength regardless of their number.

Considering the drastic impact TAD boundary variations can have on development (Lupiáñez et al., 2015), the TAD structure differences that we observed between 90 and 110 days of gestation are likely to regulate the expression of genes involved in pig muscle maturation. Notably, we found several genes with muscle-related functions in the regions that differ between overlapping group-specific TADs, including *GAP43*, *PECR* and *STIM2* for instance (Darbellay et al., 2010; Guarnieri et al., 2013; Piórkowska et al., 2017). The set of group-specific TADs is provided in Supplementary File 2.

Altogether, these results showed that, while most of the TADs were preserved when comparing samples pairwise, a subset of the TADs was exclusively and consistently detected within either the d90 or the d110 group. The difference in the insulation capacity of their boundaries during time suggests that these TADs contribute to reshaping the structural organization of the pig genome during gestation.

### 3.3 Genome Compartments Identification Revealed a Major Chromatin Remodeling During Muscle Maturity in Pig

At a higher level of organization, we investigated the segmentation of the chromosomes into A and B epigenetic compartments using the interaction matrix of each replicate. We identified 682 compartments per replicate on average (Supplementary Table 2) with a median size between 2.6 and 3.5 Mb, in the same order of magnitude than what was reported in human or mouse cells (Dixon et al., 2012; Lieberman-Aiden et al., 2009). As observed with TADs, compartment predictions were highly similar between matrices: 83.3% of the genomic regions with a prediction in each of the six samples were assigned the same compartment type in all of them consistently (either A or B six times, Figure 2 and Supplementary Figure 1), which is significantly higher than expected by chance (*p*-value < 1*e* ^−3^, permutation test). These results illustrate the high level of reproducibility between replicates and argue for a general conservation of the higher structural organization level of the genome, as previously observed in other organisms (Barutcu et al., 2015; Dixon et al., 2015; Doynova et al., 2017).

**FIGURE 4:**
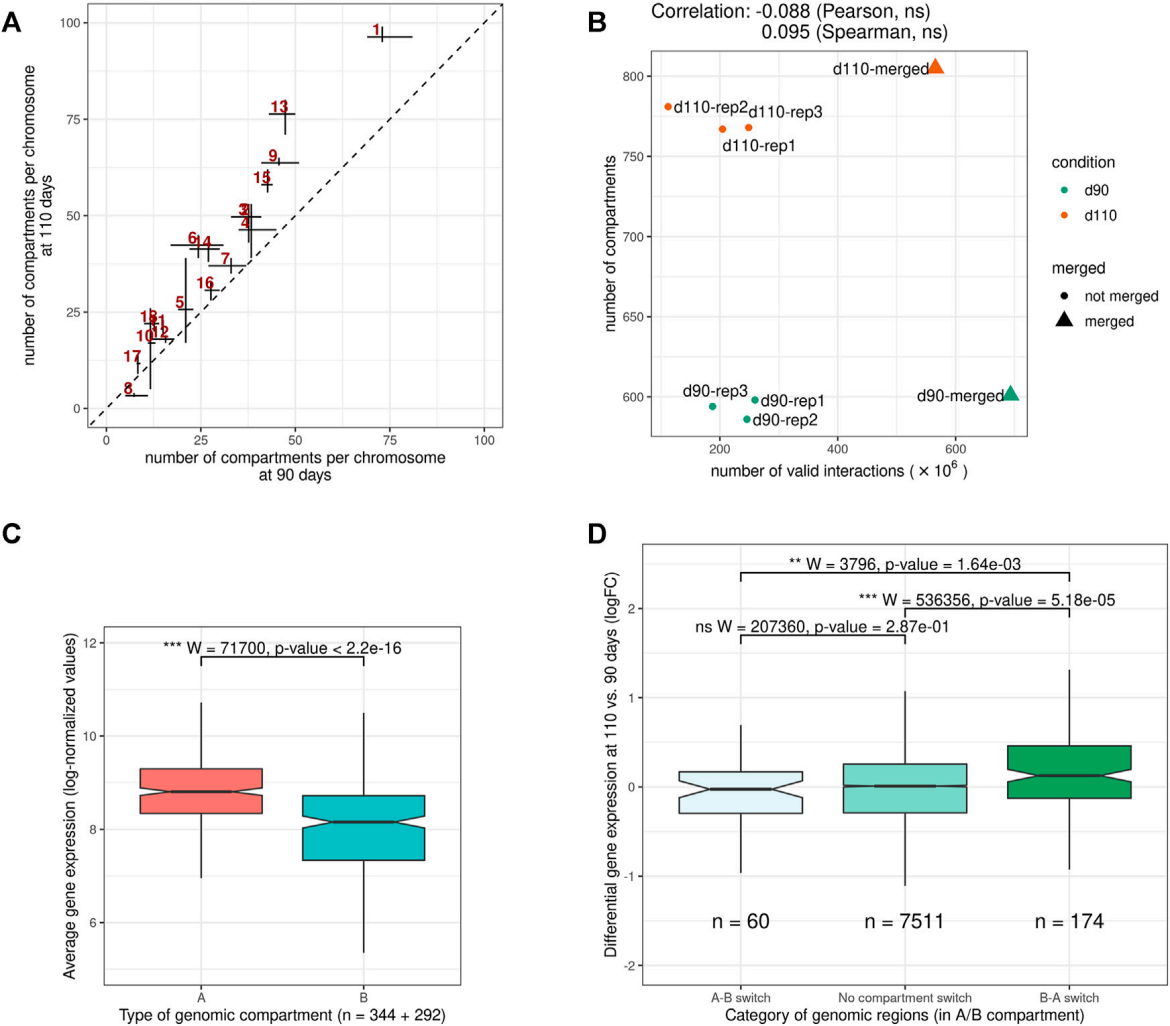
Features of A/B genomic compartments **(A)** Average number of compartments per chromosome at 90 and 110 days of gestation. The dotted line indicates *y* = *x* **(B)** Relation between the number of valid interactions in each matrix and the number of compartments. Unlike for TADs (Figure 3B), no impact was detected **(C)** Average expression of genes in A vs B compartments. Gene expression data were obtained from a previous study of fetal muscle samples at 90 and 110 days of gestation Voillet et al. (2014) **(D)** Distribution of differential expression values (logFC) for genes in genomic regions **(left)** switching from an A compartment at 90 days to a B compartment at 110 days (A-B switch) **(middle)** showing no compartment switch **(right)** switching from a B compartment at 90 days to an A compartment at 110 days (B-A switch). See also Supplementary Figures S1 and S2.

Despite this general consistency, a striking discrepancy appeared between groups. Indeed, for all replicates, d110 compartments were systematically smaller and more abundant than d90 compartments, with an increase of about 30.2% (from 593 to 772 compartments on average). A similar trend was obtained by analyzing the merged matrices (from 601 to 804 compartments for merged90 and merged110 respectively, Supplementary Table 2). This difference in the number of compartments was observed genome-wide and for both compartment types, suggesting a general fragmentation of the compartmentalization during development (Figure 4A, Supplementary Figure 1 and Supplementary Table 2). Interestingly, contrary to what was observed for TADs, no substantial correlation was detected between the total number of interactions and that of compartments (Pearson coefficient of correlation = − 0.09, *p*-value = 0.84, Figure 4B), ruling out variation in matrix density as a plausible explanation for this difference. These results support the idea of a major functional switch taking place in muscle cells during the maturity process, as already evidenced by expression networks (Voillet et al., 2014) and metabolomic analyses (Lefort et al., 2020). Moreover, they strongly suggest that the underlying regulatory program involves epigenetic modifications through a genome-wide chromatin structure remodeling.

To investigate the potential role of such remodeling, we used gene expression data from a previous study on muscle samples at 90 and 110 days of gestation (Voillet et al., 2014). In a first step, we confirmed that gene expression values were significantly higher in A vs B compartments overall (*p*-value < 2.2*e* ^−16^, Wilcoxon test, Figure 4C), as observed in other species (Lieberman-Aiden et al., 2009). Notably, the fact that consistent results were obtained from gene expression and chromosome conformation experiments that were conducted on different animals in different studies emphasizes the relevance of the data. A similar difference was also obtained comparing gene density in A vs B compartments (Supplementary Figure 2). Next, we considered genomic regions with different compartment dynamics during the maturity process–*i.e.*, whether they stay in the same compartment type, switch from A to B or from B to A–and compared their respective dynamics of gene expression between 90 and 110 days of gestation (see *A/B compartments detection*). Again, although expression and conformation data came from different animals, a slight yet significant difference was found between groups of genes in accordance with the expected results considering the gene position: genes in regions that switched from inactive (B) to active (A) compartments tend to have higher fold-change expression values than those in A-to-B switching regions, with stable regions in between (*p*-values = 1.64*e* ^−3^ for the difference between A-to-B and B-to-A switches, Wilcoxon test, Figure 4D). Altogether, these results suggest functional links between the genome-wide reorganization of the chromatin structure and the global modification of the gene expression program that was already reported during muscle maturity in pig.

### 3.4 Comparative Analysis of Hi-C Maps Identified Significantly Different Interactions Between Gestational Stages

**FIGURE 5:**
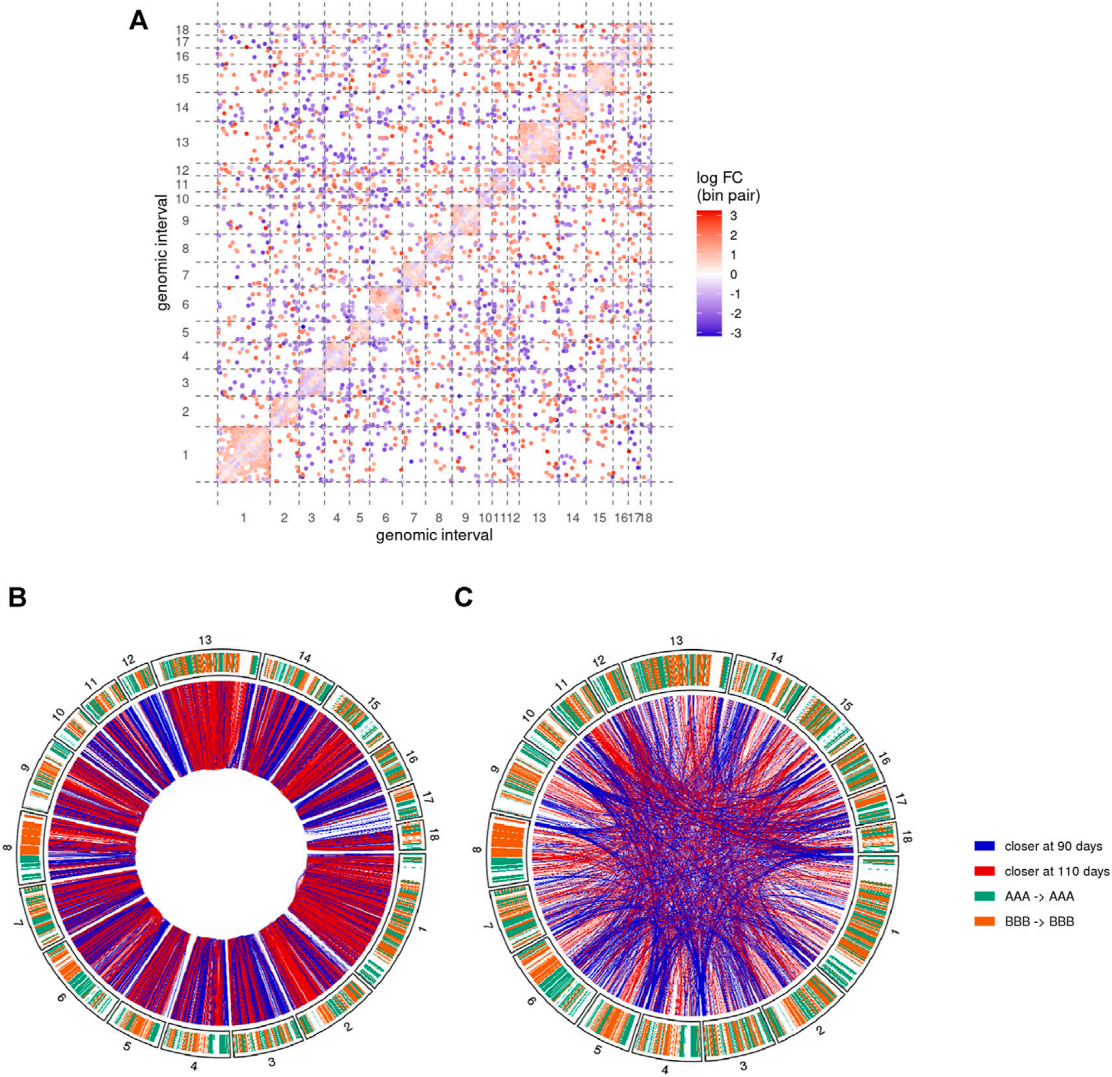
Pairs of genomic regions with differential interactions between 90 and 110 days of gestation. Results of the comparative analysis of the Hi-C matrices at 500 Kb resolution show differential interactions along the 18 assembled autosomes **(A)** Differential interaction matrix. Each dot represents a pair of genomic interval with a significantly different interaction value and its associated log-fold change value (logFC, blue-white-red gradient scale). Positive values of logFC correspond to genomic regions closer at 110 days of gestation than at 90 days (red dots). Inversely, negative values indicate regions that were closer at 90 days (blue dots). Same colors are used to display *cis* **(B)** and *trans* **(C)** differential interactions as red (positive logFC) or blue (negative logFC) connections between genomic regions (outer circle). Chromosome inner color shows the genomic segmentation into A (turquoise) and B (orange) stable compartments. See also Supplementary Figures S3 and S4.

We then performed a comparative analysis of the Hi-C matrices to identify pairs of genomic regions with significantly different interaction values between groups of samples (see *Detection of differential interactions*). This analysis led to the identification of 10,183 differential interactions between pairs of 500 Kb genomic regions. While this only represents 0.11% of the 9,262,199 tested interactions, the corresponding regions involved a substantial proportion of the genomic space across all chromosomes (Figure 5A). Among the differential interactions, 8,332 (81.8%) were *cis* interactions, *i.e.*, between two genomic regions from the same chromosome. This predominance is likely due to the fact that Hi-C matrices typically feature relatively low values for *trans* interactions, resulting in a weaker statistical power than for *cis* interactions.

About 57% of the differential interactions showed a positive log-fold change (logFC), meaning that they contain significantly more connections at 110 days than at 90 days. These regions are therefore expected to become closer together during the 90–110 days transition. Inversely, negative logFC should characterize pairs of regions that become more distant during development. Interestingly, despite a rather balanced ratio of positive/negative logFC overall, the proportion of differential interactions with positive and negative logFC was highly heterogeneous across chromosomes (Figure 5A).

### 3.5 Regions Involved in Differential *cis* Interactions Form Homogenous Blocks of Chromatin Compaction

To further investigate the genomic distribution of significantly different interactions, we first focused on *cis* differential interactions and represented them along the chromosomes depending on the sign of their logFC (Figure 5B). Although each single genomic locus could potentially be involved in differential interactions of opposite logFC signs (by moving from one region to another one for instance), we noted a general predominance of one of the signs. More precisely, out of the 3,616 distinct 500 Kb regions involved in at least one differential interaction, 2,261 of them (62.5%) have either only one type (with positive or negative logFC) of interaction or at least 10 times more interactions of one type. Interestingly, regions with such a predominance of one sign tended to cluster adjacently along the genome to form homogenous blocks of either positive or negative differential interactions (Figure 5B). For instance, chromosomes 1, 13 and the q arm of chromosome 2 were largely covered by blocks of positive logFC, while blocks of negative logFC could be found in large chunks of chromosomes 3, 12 and 14 (Figure 5B). We termed these homogenous blocks BODIs, for Blocks Of Differential Interactions, and assigned to each of them its predominant logFC sign.

We first wanted to assess the significance of this observation, considering that some of the differential interactions with the same sign were expected to involve adjacent regions just by chance, necessarily forming blocks of variable sizes. To do so, we compared the size distribution of the observed BODIs with that of artificial BODIs obtained after randomly shuffling the logFC signs of the existing interactions (see *Characterization of BODIs*). We found a significant overrepresentation of both positive and negative BODIs of size equal or greater than 2.5 Mb up to 5 Mb (p-value < 10*e* ^−3^, permutation test, Supplementary Figure 3), supporting the relevance of the observed BODIs.

Assuming that a drastic accumulation or depletion of pairwise interactions could result from variations of chromatin density, we hypothesized that positive BODIs could indicate genomic regions that undergo chromatin compaction during development. Inversely, negative BODIs would then reflect wide de-condensation events along the chromosomes. We therefore checked for consistency with the positions of A/B compartments. Interestingly, while BODIs could be found in every chromosome with a variable proportion of positive/negative BODIs, their genomic distribution in A and B compartments seemed to depend on their sign. Indeed, 58% of the genomic space in negative BODIs belonged to A compartments, while this overlap was only 30% for positive BODIs. Considering that A and B compartments occupy about the same size of the genome, this discrepancy between A and B compositions of BODIs was highly significant (p-value < 2.2*e* ^−16^, Fisher’s Exact test). Consistently, a significant difference could be observed between gene expression ratios too: genes in negative BODIs had significantly higher logFC values on average than genes in positive BODIs (p-value < 2.4*e* ^−4^, Wilcoxon test). These results support an epigenetic control of the chromatin compaction during late development in muscle cells.

### 3.6 Preferential Clustering of Telomeres at 90 Days of Gestation

We then focused on the genomic distribution of *trans* interactions genome-wide and observed an accumulation of differential interactions at the chromosome extremities, in particular with negative logFC (Figure 5C and Supplementary Figure 4). These interactions involved telomeric and sub-telomeric regions from both “q” and “p” arms of several chromosomes, providing additional support for a major reorganization of the chromosome conformation during gestation.

**FIGURE 6:**
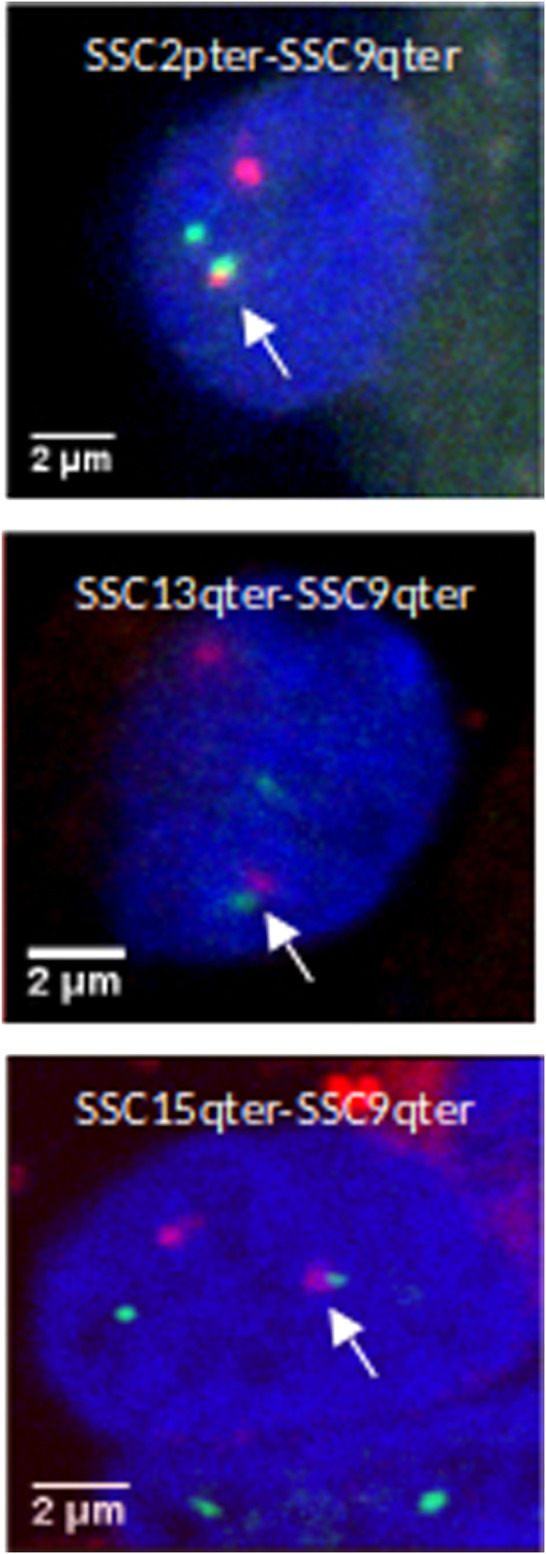
3D DNA FISH validation of preferential associations of telomeres in muscle cells. 3D images illustrating telomeric associations (SSC2pter – SSC9qter) (SSC13qter – SSC9qter) and (SSC15qter – SSC9qter) at 90 days of gestation. Maximum intensity projections of confocal image stacks are shown. SSC2p, SSC13q and SSC15q telomeres are labelled in green (Alexa 488) and SSC9qter telomere probe in red (Alexa 568). Nucleus DNA was counterstained in blue with DAPI.

In order to validate this model, three combinations of “p” or “q” telomeric associations between different chromosomes (SSC2pter – SSC9qter, SSC13qter – SSC9qter and SSC15qter – SSC9qter) were selected based on the density of differential interactions in *trans* (Figures 5C and Supplementary Figure S4) and further tested by 3D DNA FISH. The number and proportion of nuclei with telomere associations were determined for each combination at 90 and 110 days. Results are presented in Figure 6 and Table 1. All three tested combinations revealed telomere clustering at both stages. Furthermore, for each combination, we obtained significantly higher proportions of association at 90 vs 110 days (p-value = 0.02, χ2 test), confirming a consistent variation of the distance between these telomeres during late gestation (Figure 6 and Table 1).

**TABLE 1:** Numbers and proportions of nuclei with an observed association between telomeres. Proportions of nuclei harboring the probed telomeric associations in muscle cells at 90 and 110 days of gestation: SSC2pter – SSC9qter, SSC13qter – SSC9qter and SSC15qter – SSC9qter. For each association, ∼100 nuclei were analyzed. A higher percentage of association is observed at 90 days of gestation for the three tested associations.

Tested telomere interaction	Proportion of nuclei with interaction (total number of nuclei)	
	90 days of gestation	110 days of gestation
SSC9qter – SSC2pter	24% (100)	15% (100)
SSC9qter – SSC13qter	19% (99)	15% (100)
SSC9qter – SSC15qter	28% (100)	20% (97)

## 4 Discussion

### 4.1 First Insights in Porcine Muscle Genome Architecture During Late Gestation

To the best of our knowledge, the present study is the first 3D genome structure assessment performed on fetal muscle tissue in pig. The specific focus on the period of 14 and 4 days before birth, a critical gestation time for piglet survival at birth, makes our experimental design of high relevance for agronomic research (Rehfeldt et al., 2000; Foxcroft et al., 2006; Rehfeldt and Kuhn, 2006). In addition, the anatomical, physiological and genetic homologies between human and pig also make it of interest for the biomedical field (Lunney, 2007; Meurens et al., 2012). Related 3D genomics studies on muscle development were mostly performed on mouse, using *in vitro* cell cultures (Doynova et al., 2017; He et al., 2018; Zhang et al., 2020), targeting early stages (myoblasts proliferation and differentiation). Here, we focused on the maturity process of differentiated muscle fibers before birth. The closest study we know in human was performed on skeletal muscle (among other tissues) of adult subjects, not during development (Schmitt et al., 2016).

As in many studies using Hi-C assays, an obvious limitation of our experimental design is the relatively low number of biological replicates, compared for instance with differential gene expression studies. Considering the ongoing cost reduction of preparing and sequencing Hi-C libraries, we expect the average number of replicates in Hi-C studies to increase in the future, as it has been the case for RNA-seq (Rapaport et al., 2013; Liu et al., 2014). Another limitation is the presence of a female fetus among the six fetuses of the study. While this heterogeneity increased the variability in one of the groups and consequently impacted the statistical power of the comparative analysis, we still could observe many significant differences between the two stages (see the differential interaction analysis). In addition, for A/B compartments and TADs comparisons, we chose highly stringent criteria (consistently opposed predictions between groups across all samples) to ensure a low false positive rate. The consistency with gene expression data from another study (see A/B compartment switches) and DNA FISH experiments (see telomere clustering) argue for the reliability of the results and for the structural plasticity of the porcine genome during late development.

### 4.2 TAD Stability vs Variability: An Open Question

Numerous studies have led to the widespread perception that TADs are highly conserved across cell types and species (Dixon et al., 2012; Rao et al., 2014; Bonev and Cavalli, 2016; Schmitt et al., 2016). However, recent reports have highlighted the variability of the TAD organization between or within species (Eres and Gilad, 2020), including between biological replicates of the same tissue or cell line (Sauerwald et al., 2020). Several reasons can explain this heterogeneity. First, TAD variability highly depends on the nature of the samples that are being compared. As in gene expression assessment for instance, one could reasonably expect samples from functionally similar tissues to generate closer results compared with samples from unrelated tissues. The lack of available data is another obstacle to correctly assess TAD variability, even among samples from the same tissue or cell line. Indeed, due to their high experimental cost compared with other assays like RNA-seq for instance, Hi-C experiments are usually not performed on a large number of replicates. Consequently, apart from some widely used human or mouse cell lines, most of the currently available datasets only propose biological duplicates, in particular for tissue samples. Obviously, the lack of a proper and commonly accepted definition of TADs also hampers the estimation of their variability. Consistently, benchmarking studies of TAD detection methods frequently report heterogeneous results (Dali and Blanchette, 2017; Zufferey et al., 2018).

Here, we showed that, by analyzing six samples from two different development stages of the same tissue, we could survey a wide spectrum of the topological landscape. On the one hand, pairwise comparisons between replicates of the same tissue–even from different gestational stages–resulted in a majority of identical TADs, thereby supporting the idea of a stable topological landscape. Moreover, we could identify a subset of highly stable TADs that were consistently detected in all samples regardless of the group. On the other hand, only a small proportion of the TADs (less than one third) fell into this category, meaning that the vast majority could not be found in all the samples. Also, we could identify a subset of variable TADs that were consistently group-specific, potentially enabling regulatory programs of gene expression. The presence of several genes with muscle-related function in the variable regions of these group-specific TADs supports this hypothesis, and provides interesting candidates for further functional investigations. Besides transcriptional regulation, part of this TAD variability could also be due to mechanisms like DNA replication and repair (McCord et al., 2020), which are particularly active during fetus development.

Overall, due to the limited relevance of any general statement on TAD variability/stability, the main challenge is probably less about estimating how variable/stable TADs are than about identifying which TADs can reliably be considered as variable/stable. In this context, ongoing efforts in data production and analysis are providing substantial help to complete and explore the known panorama of chromatin topologies, including in farm species (Giuffra et al., 2019). As for any functionally relevant genomic feature, the capacity to distinguish stable from variable TADs is undoubtedly an important asset to decipher the molecular mechanisms underlying their formation, regulation and conservation.

### 4.3 Switching Compartments in Muscle Nuclei During Late Gestation

We confirmed several known features of A/B genome compartments related to gene density, expression, and general stability across replicates (Lieberman-Aiden et al., 2009; Barutcu et al., 2015; Doynova et al., 2017; Foissac et al., 2019). Although the median size of our compartments was in line with previous reports (Lieberman-Aiden et al., 2009; Dixon et al., 2012; Foissac et al., 2019), a decrease of the compartment size was observed at the end of gestation in our fetal samples, suggesting a fragmentation of the compartments. We observed about 3% of the genomic regions that underwent a total and consistent compartment switch considering the three replicates of each condition. These dynamic changes seem less important compared with some studies where extensive A/B compartment switches were observed. For instance, up to 25% of switches were reported in pairwise comparisons between human embryonic stem (ES) cells and mesenchymal stem cells (MSCs) (Dixon et al., 2015), 12% between epithelial and breast cancer cells (Barutcu et al., 2015), and from 8 to 21% between progenitor and differentiated myotubes (Doynova et al., 2017; He et al., 2018). However, in these studies, the switching regions were identified after merging all replicates for each condition without considering consistency across replicates. Moreover, the A/B compartments were identified at different resolutions in each study (from 40 Kb to 500 Kb). Fine changes that cannot be observed at low resolutions might be detected by using smaller bin sizes, consequently increasing the number of variable genome regions. On the other hand, high resolution analyses require a large amount of data. False positive switches are expected in genomic regions with low read coverage for instance, especially in pairwise comparisons of merged samples that do not take biological replicates into account. This could partly explain the higher percentages of switching compartments found in previous studies. Nevertheless, cell or tissue type is likely the main driver of compartment variability, as shown for TAD structures (Sauerwald et al., 2020). In Dixon et al. (2015) for instance, mesendoderm (ME) cells and MSCs showed 3.8 and 25% of switches respectively compared with their ES progenitors cells, suggesting that the more divergent the cell populations, the more important the differences in chromatin structure. In this context, while our study features a relatively low proportion of compartment switches, the consistency across replicates plus the fact that all cell populations come from the same tissue type (differentiated muscle fibers from late development stages) strongly argue for a biological significance of these results. The consistency with previously obtained gene expression results (associating opposite expression dynamics to genes in symmetrical compartment switches) further supports the role of chromatin structure on gene expression, in agreement with previously reported results in human and mouse (Barutcu et al., 2015; Dixon et al., 2015; Won et al., 2016; Doynova et al., 2017; He et al., 2018).

### 4.4 Dynamic Interacting Genomic Regions During the Maturity Process of Fetal Muscle

In this study, we could detect genome-wide dynamic changes in the chromatin structure of muscle nuclei occurring at late gestation. Specifically, we identified 10,183 differential interactions at 500 Kb resolution between the 90th and the 110th day of gestation. As noted above, considering our model of differentiated muscle fibers at two relatively close developmental stages, minor differences could have been expected. For instance, we detected much more differentially interacting regions compared with the murine myogenesis *in vitro* model (Doynova et al., 2017; He et al., 2018), where only 55 and 2,609 differential interactions were reported between myoblasts and differentiated myotubes respectively.

The differential interactions were distributed all over the genome but not homogeneously. We observed large genomic regions of adjacent differential interactions with the same dynamic behavior when comparing the two gestational ages, sometimes along entire chromosome arms. Similar results were observed on the fly genome, where higher-order clusters corresponding to each chromosome arm were organized into active and inactive clusters (Sexton et al., 2012). However, those results were not associated to dynamic changes as the fly study was focused on an exhaustive description of 3D folding features rather than on a comparison between two different conditions. This chromatin remodeling of large adjacent regions might be involved in the transcriptional and metabolic changes previously observed in fetal pig muscle (Lefort et al., 2020; Voillet et al., 2014, 2018).

Interestingly, we found that 58% of the genomic space in the negative BODIs was located in A compartments compared with only 30% for positive BODIs. To explain these results, we hypothesize that the structural and functional environment of A and B compartments may induce changes on the chromatin state of local regions located inside each compartment type. For instance, following our definition that negative BODIs are genomic regions that were closer (more condensed) at 90 days of gestation and that become farther apart at the end of gestation, we propose that those negative BODIs located on a decondensed/active environment (A compartment) follow a chromatin activation/de-condensation through development promoted by the genomic active environment.

### 4.5 Inter-chromosomal Telomeres Clustering

We found multiple dynamic associations between the telomeric regions (telomeres clustering) of several chromosomes involving either the p or the q arm. The density of interactions between telomeres decreases at 110 days of gestation. Nevertheless, 3D DNA FISH analyses do not suggest a dissociation of the clusters at the end of gestation but a higher prevalence of telomeres clustering at 90 days of gestation compared with 110 days. This indicates that telomeric regions exhibit a dynamic coordinated nuclear organization in muscle cells during late development. In fact, telomeres have been observed to display rapid movements in live human cells (Wang et al., 2008).

Interactions between telomeric regions have been widely reported in several species: preferential contacts between telomeres have been reported in fly embryonic nuclei, although these contacts were not associated with dynamic changes (Sexton et al., 2012). Another study showed that telomeric and sub-telomeric regions exhibit more frequent interactions in epithelial cells than in breast cancer cells (Barutcu et al., 2015). In this latter study, however, only intra- but not inter-chromosomal interactions were reported, meaning that some chromosomes bend to bring their extremities in contact with each other. This chromosome bending phenomenon was also reported in pig neutrophils (Mompart et al., 2013). Besides, the telomeres clustering has also been observed in yeast meiotic and quiescent cells (Yamamoto, 2014; Guidi et al., 2015; Lazar-Stefanita et al., 2017). In yeast, the telomere clustering has been associated to the formation of foci in which silencing factors concentrate, and the dynamic nature of aggregation or dissociation of these clusters has been also demonstrated (Hozé et al., 2013). Further evidences of telomere clustering have been found in mammals both in somatic cells and gametes (Solov’eva et al., 2004). For instance, in human cancer and mouse cell lines, dynamic associations and dissociations of telomere fractions were observed in quiescent cells (Molenaar et al., 2003); in human fibroblasts, telomeres were found preferentially associated in interphase nuclei than in their cycling counterparts (Nagele et al., 2001); and in pig, a strong clustering of telomeres was reported in differentiated immune cells like neutrophils and lymphocytes (Yerle-Bouissou et al., 2009).

Interestingly, in human myoblasts, long telomeres have been observed to be involved in forming chromosome loops that can affect the higher order chromatin structure and gene expression (Robin et al., 2014). It was proposed that telomere length-dependent long-range chromosomal interactions may repress (or enhance) gene expression by respectively silencing (or activating) those genes close to the telomere when telomeres become shorter with cellular aging. Besides, the SMARCA4 subunit of the SWI/SNF complex, which has a potential role in tissue-specific gene regulation during embryonic development, has been suggested to play a role in three-dimensional organization of telomeric regions (Barutcu et al., 2016). In addition, the ATPase subunit of this same SWI/SNF complex has been found to be required for the formation of inter-chromosomal interactions contributing to changes in gene positioning during myogenesis and temporal regulation during myogenic transcription (Harada et al., 2015). Our finding of inter-chromosomal clustering of telomeric regions during late gestation, together with the aforementioned studies related to telomere associations, raise the possibility of a specific dynamic mechanism of gene expression regulation in fetal muscle cells through temporal formation-disruption of telomere clusters.

In conclusion, we found major changes of the 3D genome structure during the establishment of muscle maturity at late gestation. These changes occur concomitantly with previously reported modifications of the transcriptional program, between 90 and 110 days of gestation. The topological reorganization that we observed implies structures of various scales, including individual interactions, TADs and large BODIs. The proportion of the genome that was impacted depended on the nature of the modification. Some of the changes, such as the fragmentation of the genomic A/B compartments, impacted most of the chromosomes, while others, such as the telomere clustering, involved specific regions. The amplitude of these modifications is particularly striking considering that two close fetal development stages were compared. This suggests that topological changes of the 3D genome of organized tissues could be as remarkable as changes observed during cell differentiation and cell commitment.

## Data Availability

Animals and samples metadata have been registered at BioSamples (https://www.ebi.ac.uk/biosamples) and are available using the accession ID SAMEA7390788. Experimental protocols have been deposited at the FAANG DCC: https://data.faang.org/api/fire_api/samples/INRAE_SOP_pig_muscle_tissue_sampling_20200812.pdf (sampling) and https://data.faang.org/api/fire_api/assays/INRAE_SOP_Hi-C_pig_muscle_tissue_20200812.pdf (Hi-C libraries). Raw sequencing data from Hi-C experiments have been uploaded to the ENA under the accession PRJEB40576 (ERP124229) and to the FAANG DCC along with the associated metadata, as per the FAANG consortium agreement (https://www.faang.org/data-share-principle). Hi-C matrices, TADs and A/B compartments generated in this study have been made available at: https://doi.org/10.15454/DOMEHB and http://www.fragencode.org/pig3Dgenome.html.
