# Supplementary material for: Major Reorganization of Chromosome Conformation During Muscle Development in Pig
Source: Front Genet. 2021 Oct 5;12:748239. doi: 10.3389/fgene.2021.748239 (PMC8523936; doi:10.3389/fgene.2021.748239)
Supplement: Supplementary file 1 [file DataSheet1.PDF]

Supplementary Material

1 SUPPLEMENTARY FILES

- **Supplementary file 1.** Stable TADs in BED format (tsv).

- **Supplementary file 2.** Group-specific TADs in BED format (tsv).

2 SUPPLEMENTARY TABLES AND FIGURES

2.1 Figures

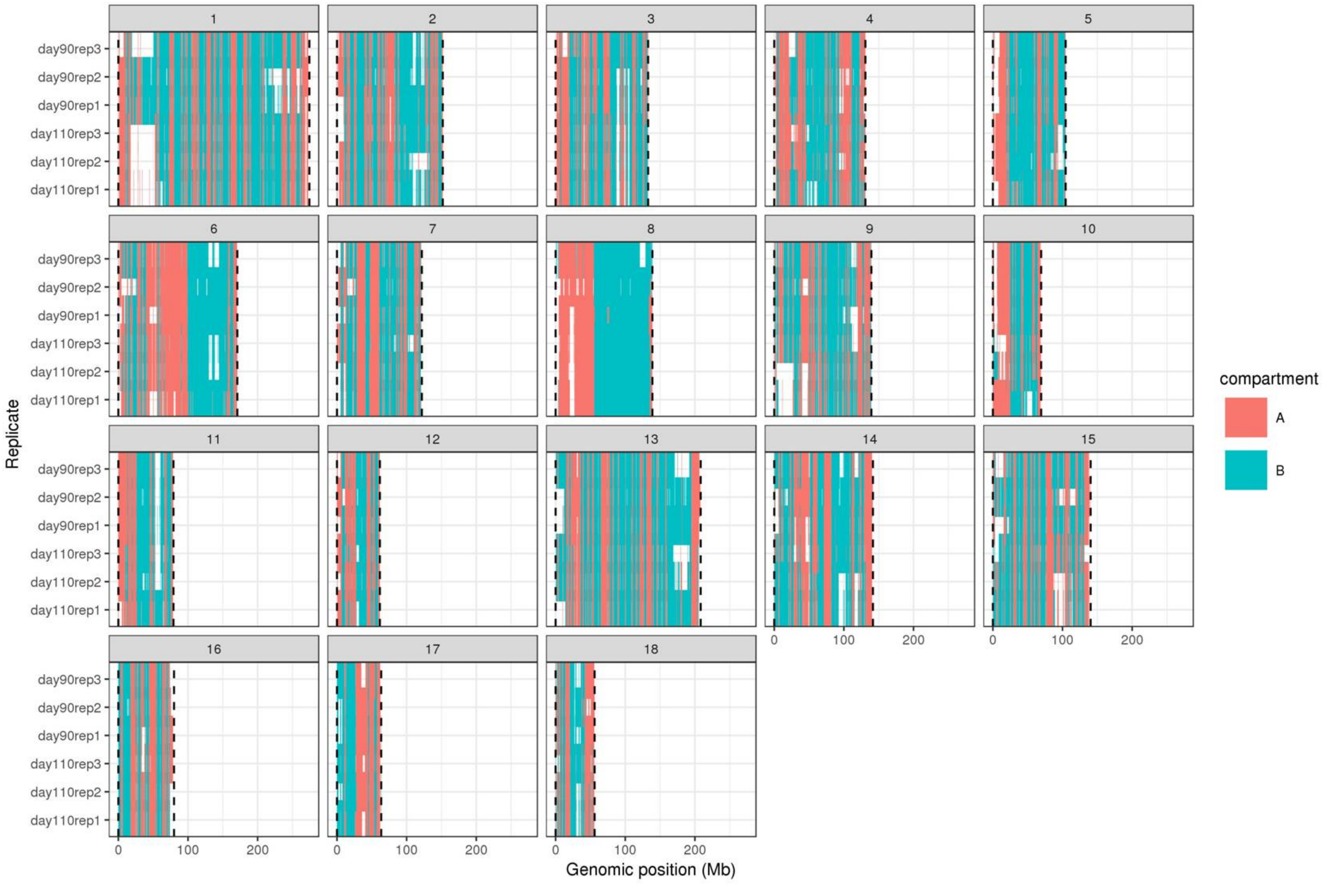

Figure S1. Distribution of Hi-C A and B compartments along each chromosome for each replicate. Genome-wide overview of compartment labels per 500 Kb bin. A general consistency can be observed across replicates. Dotted lines delimit the beginning and the end of each chromosome. White regions are devoid of any called compartment.

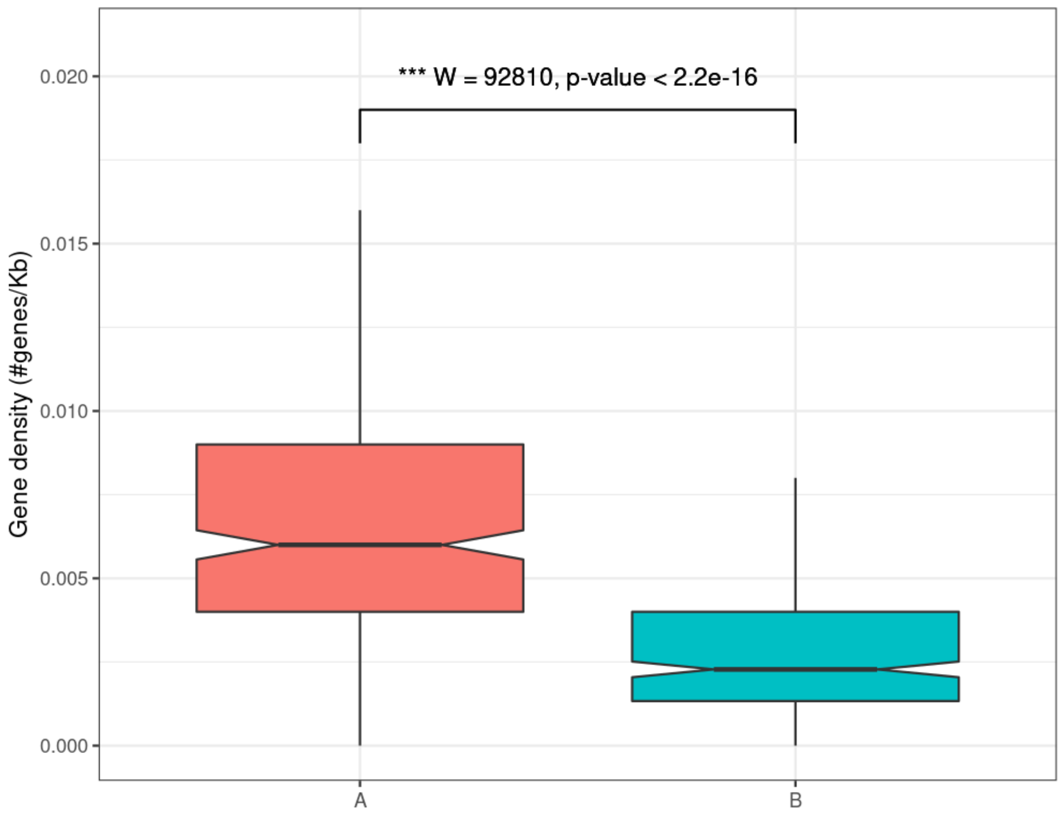

Figure S2. Average gene density in A/B compartments. Average number of distinct "gene_id" identifiers were computed for each A and B compartment using the reference annotation ENSEMBL v98.

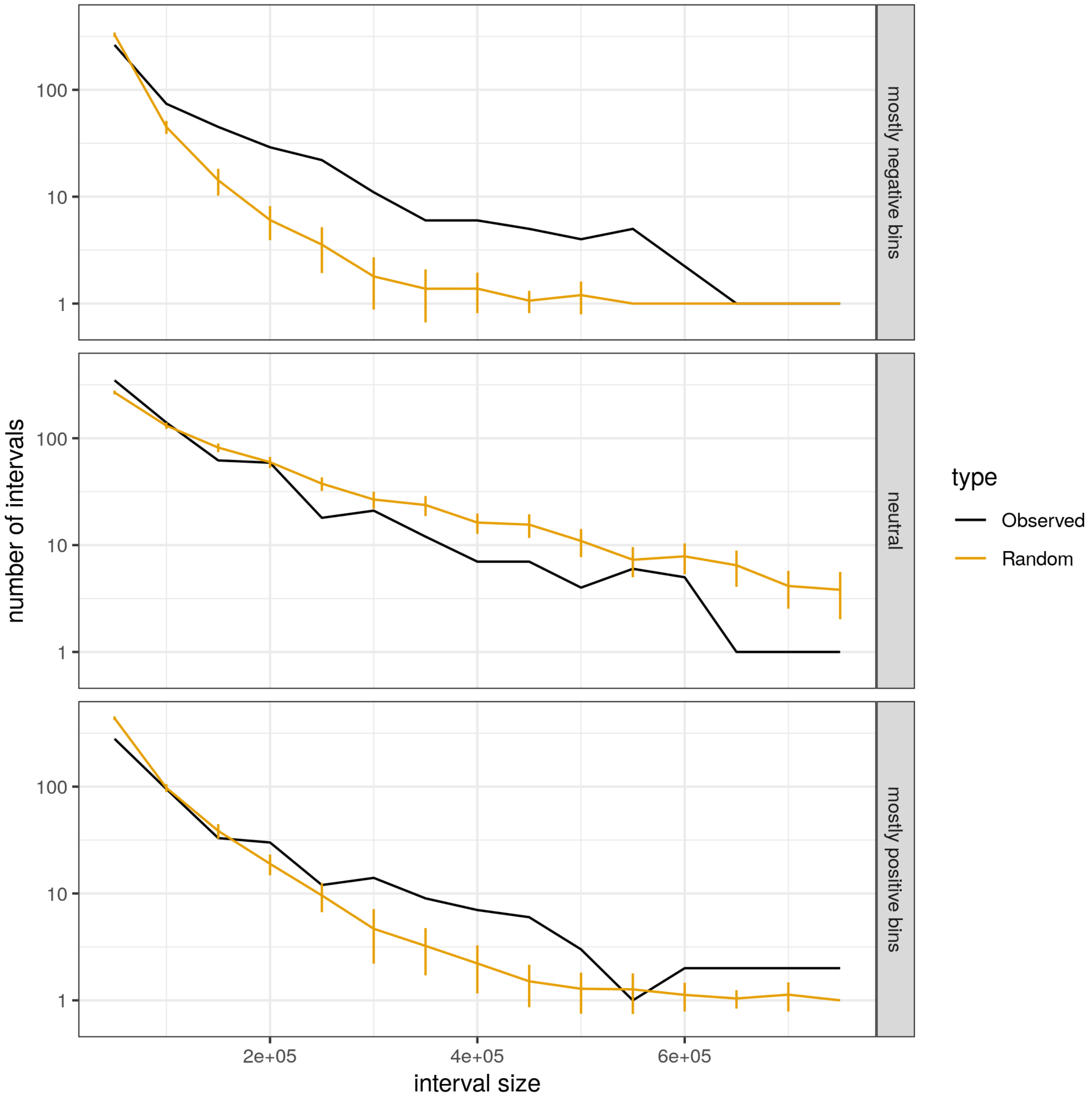

Figure S3. Enrichment of large Blocks Of Differential Interactions (BODIs). Size distribution (x axis, size in bp) of actual (observed, black line) and randomly generated (expected by chance, yellow line) BODIs ("intervals") with a prevalence of negative logFC (-1), positive logFC (1) or a mixture of both (0). Randomly shuffling logFC values results in a sharp decrease of long positive and negative BODIs compared to the observed quantities, with a significant drift for BODIs of 5 consecutive genomic bins of 500 Kb (2.5 Mb) or larger. Loss of significance after 10 bins can be explained to the low number of observed BODIs larger than 5 Mb.

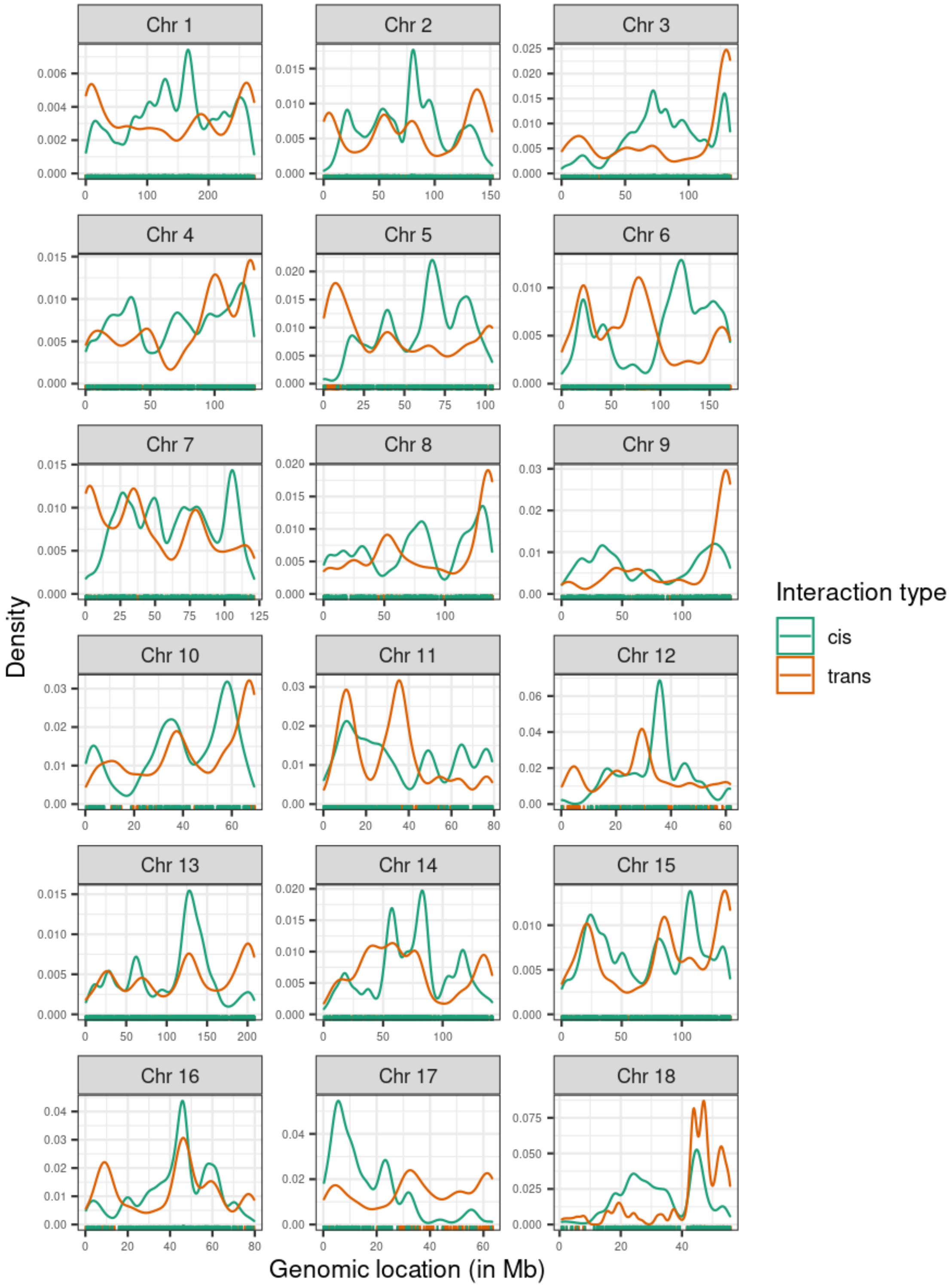

Figure S4. Density of *cis* and *trans* differential interactions along the chromosomes. Comparing the density (y-axis) of both types of differential interactions along chromosomes using a normalized relative scale (x-axis) allows to highlight the preference for trans interactions to involve telomeric regions of several chromosomes.

2.2 Tables

Organism name in SRA	Corresponding specimen in SRA	Sex	Animal age
F1039-90	Rep1-90-160216	Male	90 days of gestation
F1044-90	Rep2-90-160223	Male	90 days of gestation
F1043-90	Rep3-90-160308	Male	90 days of gestation
F0036-110	Rep1-110-160307	Female	110 days of gestation
F1022-110	Rep2-110-160308	Male	110 days of gestation
F1031-110	Rep3-110-160223	Male	110 days of gestation

Table S1. Description of animals and corresponding specimen as deposited in BioSamples. `https://www.ebi.ac.uk/biosamples` – Accession ID SAMEA7390788.

Sample	# read pairs (library size)	# mapped reads (all chrom.)	# valid pairs (matrix size)	# valid pairs in *cis* (same chr)	# valid pairs in *trans* (inter chr)	# TADs	# A/B compart.
Rep1 d90	654,909,642	907,023,732	259,576,433	149,610,945 (57.64%)	109,965,488 (42.36%)	1,585	598
Rep2 d90	685,369,147	922,065,942	245,848,286	126,078,924 (51.28%)	119,769,362 (48.72%)	1,432	586
Rep3 d90	489,704,084	683,027,224	187,651,263	117,036,377 (62.37%)	70,614,886 (37.63%)	1,500	594
Merged90	1,829,982,873	2,512,116,898	693,075,982	392,726,246 (56.66%)	300,349,736 (43.34%)	1,985	601
Rep1 d110	494,332,893	725,809,590	204,467,368	96,033,865 (46.97%)	108,433,503 (53.03%)	1,013	767
Rep2 d110	476,204,172	604,136,062	112,221,970	63,748,426 (56.81%)	48,473,544 (43.19%)	951	781
Rep3 d110	646,908,804	893,140,392	248,891,148	138,254,245 (55.55%)	110,636,903 (44.45%)	1,393	768
Merged110	1,617,445,869	2,223,086,044	565,580,486	298,036,536 (52.70%)	267,543,950 (47.30%)	1,705	805

Table S2. Statistics of Hi-C data per replicate. For each sample (biological replicate) and for the total by group metrics at different levels of the data analysis are reported: total number of sequenced read pairs (library size), number of mapped pairs (on the entire *Sscrofa* 11.1 genome, including all chromosomes and scaffolds), number of valid pairs (number of interactions per matrix, only for chromosomes 1-18), number and proportion of valid pairs in *cis* and *trans*, number of TADs and number of A/B compartments. Note that TADs and compartments have been predicted in each "merged" matrix independently from the others, so their numbers are not equal to the sum of those from the replicates.

Table S3. Key resources table.

Reagent or resource	Source	Identifier
**Chemicals, peptides, and recombinant proteins**		
Phosphate-buffered saline (PBS)	Gibco	10010-015
Dulbecco's Modified Eagle's Medium (DMEM) with glutamax	Fisher scientific	11574516
Formaldehyde 37% F8775-4×25ML	Sigma-Aldrich	50-980-494
Glycine	Sigma-Aldrich	50046
Protease inhibitor	ThermoFisher Scientific	A32965
4',6-diamidino-2-phénylindole (DAPI)	Vector Laboratories	H-1200
Phalloidin - TRITC	Sigma	P 1951
Sodium Dodecyl Sulfate (SDS)	Thermo Fisher Scientific	28364
Triton X-100	Sigma-Aldrich	T8787
HindIII 100 U/μl	NEB	R0104M
biotin-14-dCTP (0.4 mM)	Fisher/Invitrogen	10022582
DNA Polymerase I, Large (Klenow) Fragment (50 U/μl)	NEB	M0210M
T4 DNA ligase 20,000 U/μl	NEB	M0202M
10X T4 DNA ligase buffer (3 U/μl)	NEB	B0202S
BSA (20 mg/ml)	NEB	B9000S
dNTP solution set (10 mM)	Fisher/Ambion	AM8200
Proteinase K (20 mg/ml)	Fisher/Thermo	EO0491
CleanPCR magnetic beads	Labclinics	CPCR-0050-D
T4 DNA polymerase	NEB	M0203L
bovine serum albumin (BSA) (714 ng/ml)	Sigma	A6003-10G
10X NE Buffer 2	NEB	B7002S
NheI-HF 20 U/μl	NEB	R3131L
Triton ×100	Sigma	T87-87
ChromaTide Alexa Fluor 568-5-DUTP	Fisher scientific	10413222
ChromaTide Alexa Fluor 488-5-DUTP	Fisher scientific	10173952
Biotin 6 dUTP	Roche	11 093 070 910
Microspin G50	Dutscher	27-5330-01
Formamide	Sigma	47671-1L-F
Porcine Hybloc™ DNA PHB (1 mg/ml)	Applied Genetics Laboratories	
Ultra-pure Salmon Sperm DNA	Invitrogen	15632011
Slide superfrost plus_ THERMO SCIENTIFIC	LABELIANS (CML)	LSFPLUS
Hybridation solution	Sigma	H7782-6mL
SSC 20X	Fisher scientific	11598976
**Critical commercial assays**		
Nextera Mate Pair Sample Prep Kit – Box2 (wash solutions)	Illumina	15038732
M-280 streptavidin magnetic Dynabeads	Thermo Fisher Scientific	11205D
Mix (TruSeqNano DNA library prep)	Illumina	20015964, 20015960, 20015961
Bioprime DNA labelling kit	Invitrogen	18094-011

Reagent or resource	Source	Identifier
**Deposited data**		
Expression dataset of fetal muscle	Voillet et al. (2014)	GEO: GSE56301
Hi-C data	This study	ENA https://www.ebi.ac.uk/ena/ – Accession ID PRJEB40576 (ERP124229) add additional repos
Intermediate data	This study	Data INRAE – DOI: https://doi.org/10.15454/DOMEHB
**Experimental models: organisms/strains**		
Sus scrofa (European Large white breed)	N/A	BioSamples https://www.ebi.ac.uk/biosamples – Accession ID SAMEA7390788
**Oligonucleotides**		
Fwd1: 5' TCTGGGCAGGTCACTCATT 3'	Foissac et al. (2019)	N/A
Fwd2: 5' TCTCGGGATGCTGAGTGTTT 3'	Foissac et al. (2019)	N/A
Rv1: 5' AAACACTCAGCATCCCGAGA 3'	Foissac et al. (2019)	N/A
**Recombinant DNA**		
Porcine Bacterial artificial clones (BACs) PigI-370D12, PigI-441D12, PigI-564B6, PigI-39F7, PigI-899B10	CRB-Anim, INRA, 2018. Biological Resource Centres for domestic animals of AgroBRC, doi: http://doi.org/10.15454/1.5613785622827378E12	N/A

Reagent or resource	Source	Identifier
**Software and algorithms**		
HiC-Pro v2.9.0	Servant et al. (2015)	http://nservant.github.io/HiC-Pro/
Bowtie 2 v2.3.3.1	Langmead and Salzberg (2012)	http://bowtie-bio.sourceforge.net/bowtie2/index.shtml
ICE	Imakaev et al. (2012)	https://github.com/mirnylab/hiclib-legacy
Juicer v1.5.3	Durand et al. (2016)	https://github.com/aidenlab/juicer
bedtools v2.26.0	Quinlan (2014)	https://github.com/arq5x/bedtools2
FIMO v.4.11.1	Grant et al. (2011)	http://meme-suite.org/doc/fimo.html
R (v3.3.3, v4.1.0, v4.1.1)	R Core Team (2017)	https://www.R-project.org/
R / Bioconductor package **HiTC** v1.18.1	Servant et al. (2012)	http://www.bioconductor.org/packages/release/bioc/html/HiTC.html
R / Bioconductor package **csaw** v1.8.1	Lun and Smyth (2016)	https://www.bioconductor.org/packages/release/bioc/html/csaw.html
R / Bioconductor package **edgeR**	Robinson et al. (2010)	http://www.bioconductor.org/packages/release/bioc/html/edgeR.html
R / Bioconductor package **hicrep** v1.6.0	Yang et al. (2017)	http://www.bioconductor.org/packages/release/bioc/html/hicrep.html
R package **tidyverse**	Wickham et al. (2019)	https://CRAN.R-project.org/package=tidyverse
R package **data.table**	N/A	https://cran.r-project.org/package=data.table
Primer3 software	http://primer3.sourceforge.net/	N/A
NEMO	Iannuccelli et al. (2010)	N/A
Volocity v6.0	Perkin Elmer	N/A
Own analysis scripts	https://forgemia.inra.fr/nathalie.villa-vialaneix/pighic	N/A

**Other**		
cell strainer (70 μm)	Greiner bio-one	542070
Superfrost Plus glass slides Thermo Scientific	VWR	630-0951

3 SUPPLEMENTARY METHODS

3.1 Animals and samples

For Hi-C and FISH experiments, *longissimus dorsi* fetal porcine muscle samples were collected from the European Large White (LW) breed (F1 ♂LW x LW♀). Specifically, three 90 days gestation male littermates and three 110 days gestation (two male littermates and one female) were used for Hi-C assays. For FISH experiments, muscle samples were collected from different fetuses (one at 90 days gestation and one at 110 days) of those in which Hi-C experiments were performed. All the fetuses used in this study were obtained by caesarean after euthanasia of healthy wild type sows and fetuses. No special breeding conditions (feeding, housing ...) were applied.

The experimental design was approved and authorized by the ethical committee (No. 84) in animal experimentation of the French Ministry of National Education, Higher Education, and Scientific Research (authorization No. 02015021016014354). The experiment authorization number for the experimental farm GenESI (Genetics, testing and innovative systems experimental unit) is A 17 661. The procedures performed in this study and the treatment of animals complied with European Union legislation (Directive 2010/63/EU) and French legislation in the Midi-Pyrénées Region of France (Decree 2001-464). All the details about the animals and samples have been registered in the BioSamples public repository (`https://www.ebi.ac.uk/biosamples`) in agreement with the FAANG best practices guidelines (`https://www.faang.org/data-share-principle`) and are available using the accession SAMEA7390788.

3.2 3D DNA FISH experiments

3.2.1 Cells and probes preparation

Fetal muscle tissue was obtained from the *Longuissimus dorsi* muscle of 90- and 110-days of gestation Large White (LW) pig and prepared as described in Lahbib-Mansais et al. (2016); Marti-Marimon et al. (2018). Stored muscle fibre packets were permeabilised for 5 to 8 min in cytoskeleton extraction buffer (100 mM NaCl, 300 mM sucrose, 3 mM MgCl2, 10 mM PIPES pH 6.8) containing 0.5% Triton X 100 and then fixed in cold 4% paraformaldehyde for 5 min. After washing in cold PBS, muscle packets were manually dilacerated directly on Superfrost glass slides (CML, Nemours, France) to isolate individual fibres, and air-dried before adding DNA probes for in situ hybridization. Bacterial artificial clones (BACs) containing specific subtelomeric sequences of porcine chromosomes 2, 9, 13 and 15 were chosen as selected by Mompart et al. (2013): SSC2p (PigI-370D12), SSC9q (PigI-441D12, PigI-564B6), SSC13q (PigI-39F7) and SSC15q (PigI-899B10). These BACs were isolated from a porcine BAC library (CRB-Anim, INRA, 2018. Biological Resource Centres for domestic animals of AgroBRC, doi: `http://doi.org/10.15454/1.5613785622827378E12`). For multiple-label experiments, approximately 120 ng of each BAC DNA was random-priming labelled directly by incorporation of dUTP Alexa Fluor (488 or 568) or indirectly with Biotin-6-dUTP detected by immuno-FISH (Bioprime DNA labelling kit, Invitrogen, Cergy Pontoise, France). Three combinations of p or q telomeres probes of different pairs of chromosomes: (SSC2qter – SSC9qter), (SSC13qter – SSC9qter) and (SSC15qter – SSC9qter) were chosen to test their rate of association as suggested by Hi-C.

3.2.2 3D DNA Fluorescence In Situ Hybridization

3D DNA FISH experiments were conducted as described in Lahbib-Mansais et al. (2016) with slight modifications. Probes were resuspended in hybridization buffer (50% formamide, 10% dextran sulphate, 2 mg/ml BSA, 2× SSC) at a final concentration of 110 ng/μl. Nuclear DNA of fibers and probes were simultaneously heat-denatured at 74 °C for 7 min on the slide and then incubated overnight at 37 °C in a DAKO hybridizer. Post-hybridization washes were then performed with gentle agitation, first twice in 2×

SSC at 40°C for 6 min, then in 2× SSC, 50% formamide pH 7.0 at 40°C for 6 min, and finally twice for 10 min in 2× SSC, then in PBS at RT. When a biotin labelled probe was used, biotins were detected with streptavidin Alexa 568 or 488 at a final concentration of 5 μg/ml for 1 hour at RT.

3D acquisitions were performed at the T.R.I. Genotoul (Toulouse Réseau Imagerie, `http://trigenotoul.com/en`) imaging core facility in Toulouse (France). Image stacks were collected using a Leica SP8 confocal microscope (Leica Instruments, Heidelberg, Germany) equipped with an oil immersion objective (plan achromatic 63× N.A. $= 1.4$). The Z-stacks (around 80 confocal planes per capture) were acquired at 1024 × 1024 pixels per frame using an 8-bit pixel depth for each channel at a constant voxel size of 0.06 × 0.06 × 0.3 μm.

3.2.3 Telomere association analysis

Images were analyzed with specific software NEMO (Iannuccelli et al., 2010), distributed under the creative commons license that can be freely downloaded from `https://forge-dga.jouy.inra.fr/projects/nemo`. Segmentations and 3D measurements between signals (center-to-center distance) were done as described in Lahbib-Mansais et al. (2016). Euclidean distances were computed with respect to the x, y and z resolutions. Given the resolution on the z axis, at least three pixels corresponding to 0.9 μm (0.3 × 3) were required for a high resolution between two separate signals; consequently, 1 μm was chosen as the upper cut-off for associated signals. For each combination of telomeres, nuclei were only analyzed when 4 signals (corresponding to the chosen telomeres of 2 chromosomes) were present. "Associated" signals were considered when they are separated by a distance (d) $\leq 1\mu$m, as done in Lahbib-Mansais et al. (2016). We determined for each combination of telomeric pairs how many nuclei were found associated among about 100 observed nuclei.

Significance of the difference in association for a telomeric pair between d90 and d110 was assessed using a χ^2 test to compare the two following hypotheses

- H_0: $\mathbf{1}_{\text{associated}} \sim \mu_j$, where μ_j is the fixed effect of telomeric pair j,
- H_1: $\mathbf{1}_{\text{associated}} \sim \mu_j + \nu_k$, where ν_k is the fixed effect of condition $k \in \{\text{d90, d110}\}$,

both fitted with a generalized linear model of the binomial family.

3.3 Hi-C experiments

3.3.1 Hi-C protocol

Hi-C experiments were performed as previously documented (Foissac et al., 2019), with slight modifications to adapt the Hi-C experiments and libraries to fetal muscle tissues.

More precisely, muscle samples from 90- and 110-day post coitum (p.c.) fetuses (3 replicates per group), were frozen in isopentane cooled with liquid nitrogen and stored at −80°C until needed. For each experiment, around 1.5 g of frozen stored muscle were thawed at Room Temperature (RT) and dissected with scalpel blades to obtain a homogenate of mushed muscle. Dissected tissue was washed in phosphate-buffered saline (PBS) to remove blood. Nuclei were disaggregated by rubbing (pipetting up-down many times), filtered through a cell strainer (70 μm) and centrifuged at 1200g 5 min to get a high yield of cells. Pellet was resuspended in 3 ml Dulbecco's Modified Eagle's Medium (DMEM) with glutamax (1% formaldehyde) and incubated 10 min at RT. To quench fixation, 0.125 M final glycine was added 5 min at RT, then cooled 5 min on ice. After 5 min centrifugation at 1200 g, pellet was washed in ice-cold PBS (with protease inhibitors). For each Hi-C experiment, 5 million cells were resuspended in 0.05% Sodium Dodecyl Sulfate (SDS) and incubated 10 min at 62 °C. To quench the SDS, 0.1% final Triton X-100 was added for 15 min at 37 °C, then 100U of HindIII in 25 μl of 10X NEbuffer 2.0 were

added to digest overnight at 37 °C on the wheel. Fifty μl of fill-in master mix (200 nMdATP, dGTP, dTTP, biotin-14-dCTP, 50U Klenow) were added and incubated at 37°C 1 hour on the wheel in order to fill overhangs and obtain blunt ends. The enzyme was inactivated at 62 °C for 20 min and 900 μl of ligation mix (1.3X T4 DNA ligase reaction buffer, 1.1% Triton X-100, 130 ng/ml BSA, 2000U T4 DNA ligase) were added and incubated 1 hour at RT and then overnight at 4 °C on the wheel. Proteins were degraded with 50 μl of Proteinase K (20 mg/ml) and 120 μl of 10% SDS at 55 °C for 30 min, then with 130 μl of NaCl 5M at 68 °C overnight. DNA was precipitated with 1.6 volume of 100% ethanol and 0.1 volume of 3M sodium acetate (pH5.2) at -80 °C 15 min, then centrifuged at 4 °C (15400rpm, 10 min). The pellet was resuspended in 70% ethanol, centrifuged at 4 °C (15400rpm, 5 min) and dissolved in nuclease-free water (20 min at 37 °C). To desalt and purify DNA, 1.8 volume of CleanPCR magnetic beads were added and incubated for 5 min. After washing with 80% ethanol (30 s, twice) and letting dry for 3 min, the beads were resuspended in TE buffer solution (10:0.1,Tris 10 mM pH8.0, EDTA 0.1 mM). To remove non-ligated biotinylated DNA, 28 μl of T4 DNA polymerase mix reaction (714 ng/ml bovine serum albumin (BSA), 5.3X NE Buffer 2, 357nM dATP, 357nM dGTP and 30U T4 DNA polymerase) were added and incubated at 12 °C for 90 min. The reaction was stopped by adding 2 μl of 0.5M Ethylenediaminetetraacetic acid (EDTA) and heating 20 min at 75 °C. Then DNA was purified with magnetic beads as explained before and resuspended in TE (10:0.1).

3.3.2 Hi-C Quality controls

After DNA digestion with HindIII, and filling-ligation of the digested ends, the HindIII target site disappears and a NheI restriction site is created instead. To check the efficiency of the Hi-C assays, PCR were performed around one HindIII restriction site with two forward primers (Fwd1: 5' TCTGGGCAGGTCACTCATT 3'; Fwd2: 5' TCTCGGGATGCTGAGTGTTT 3'; product size = 425 bp). A reverse primer combined with Fwd1 was used as a control (Rv1: 5' AAACACTCAGCATCCCGAGA 3'; product size = 465 bp). In Hi-C, some religation events allow switching the sense of one DNA fragment and PCR amplification with the two forward primers. The PCR amplification products from the couple of forward primers were digested either with HindIII or NheI (product sizes = $201 + 215$ bp). In control tubes (no filling of digested ends), HindIII should cleave the PCR products while NheI should not. In Hi-C tubes, NheI should cleave most of the PCR products while HindIII should cleave only a small fraction.

3.3.3 Hi-C library production

1.4 μg of DNA from the Hi-C experiments were fragmented with a Covaris machine. Then, 0.55 volumes of CleanPCR magnetic beads were added to the fragmented DNA to select fragments < 600 bp (5 min incubation and keeping the supernatant), and 0.7 volumes of beads were added again (5 min incubation and removing supernatant) to remove fragments < 200 bp. Then beads were washed with 80% ethanol and DNA was recovered with Resuspension Buffer. To purify biotinylated DNA, 1 volume of M-280 streptavidin magnetic Dynabeads was added and after 15 min incubation, the supernatant was removed and the beads were washed 4 times with beads wash buffer (Nextera Mate Pair Preparation Kit, Illumina) and twice with Resuspension buffer. From this point, all steps were performed while DNA remained attached to the beads. To repair DNA breaks, 60 μl of water and 40 μl of End Repair Mix 2 (TruSeqNano DNA library prep, Illumina) were added and incubated 30 min at 30 °C, and then beads were washed as explained before. To allow the adapters ligation, an "A" nucleotide was added to the 3' ends by adding 17.5 μl of water and 12.5 μl of A-Tailing Mix (TruSeqNano DNA library prep, Illumina) and incubating 30 min at 37 °C and then 5 min at 70 °C to inactivate the enzyme. To ligate the adapters to the DNA extremities, 2.5 μl of Resuspension Buffer, 2.5 μl of DNA Ligase Mix and 2.5 μl of DNA Adapter Index (TruSeqNano DNA library prep, Illumina) were added (10 min incubation at 30 °C, then 5 μl of Stop ligation Buffer) and then beads were washed as before. DNA was amplified by 12 PCR cycles (15 sec at 98 °C – 30 sec at 60 °C –

30 sec at 72 °C) by resuspending beads in 50 μl of PCR mix (25 μl Enhanced PCR mix, 5 μl PCR primer Cocktail and 20 μl water, TruSeqNano DNA library prep, Illumina). To recover DNA from the beads, 0.6 volumes of CleanPCR magnetic beads were added and incubated 5 min, and then washed twice with 80% ethanol, resuspended in 30 μl of Resupension Buffer and after placing in a magnetic rack, supernatant containing the libraries was recovered. Libraries size was controlled with the Fragment Analyzer (FA) and quantified by qPCR. In addition, an aliquot was digested by using the NheI and HindIII enzymes to verify if selected fragments are the ones containing the filled-in biotinylated religation sites as done in Belton et al. (2012). Libraries were sequenced in pool in one HiSeq3000 lane to validate their quality. For depth sequencing, the pool was paired end (PE) sequenced in 11 lines of a HiSeq3000 (reads size = 150 bases), producing from $\sim$ 476 M to 685 M read pairs per library in total (see Supplementary Table 1).

3.4 Hi-C data analysis

3.4.1 Pipeline overview

The 3,447,428,742 Paired-End reads were processed as previously reported (Foissac et al., 2019). In brief, the pipeline mainly combines existing software: HiC-Pro v2.9.0 (Servant et al., 2015) to map the reads (with Bowtie 2 v2.3.3.1 (Langmead and Salzberg, 2012)) and obtain the contact matrices, ICE to correct experiment biases between bins within each matrix (within normalization) (Imakaev et al., 2012), HiTC v1.18.1 for exploratory analysis (Servant et al., 2012), Juicer v1.5.3 for TAD finding (Durand et al., 2016).

Analysis scripts have been made available at `https://forgemia.inra.fr/nathalie.villa-vialaneix/pighic`. Some intermediate data (individual and merged matrices, TADs, compartments, ...) have also been made available at `https://doi.org/10.15454/DOMEHB`.

3.4.2 Mapping

The full dataset of reads was mapped on Sscrofa11.1. First, during the "global alignment", both reads of each pair were mapped independently in single-end (SE) mode with bowtie2 using the full read sequences. Due to their chimeric nature, many reads (30-40%) did not directly map on the genomic sequence over their entire length. These chimeric reads were trimmed in order to remove the portion beyond the ligation junction (religation sequence: AAGCTAGCTT), and then re-mapped during a "local alignment" step, still in SE mode. Global and local alignments use bowtie2-2.3.3.1-linux-x86_64 and bowtie2 options via HiC-Pro v2.9.0: `--very-sensitive -L 30` (global) or `-L 20` (local); `--score-min L,-1,-0.1` (global) or `L,-0.6,-0.2` (local); `--end-to-end`; and `--reorder`. From 46.2 to 73.7% of the previously unmapped reads were successfully mapped and therefore retrieved after trimming. Singletons (reads for which the "mate" could not be mapped: $\sim$14% of the initial material) were discarded, resulting in a total of 2,367,601,471 read pairs (68.7% of the initial material).

3.4.3 Detecting valid read pairs

Mapped read pairs were classified into valid and invalid pairs. The valid pairs are those for which the sum of distances between each read 5' extremity and the closest HindIII site downstream fits within the range of the expected molecular size distribution of the library, therefore requiring both reads to map near a HindIII restriction site. The distribution of these genomic distances (read1-to-HindIIIsite1 + read2-to-HindIIIsite2) was estimated during the quality control step and used to define the threshold values of the accepted range: from 20 bp to 1 Kb. Invalid pairs with a fragment size outside of this range were therefore discarded by providing HiC-Pro with the parameters `-i 20` (`MIN_INSERT_SIZE`) `-I 1000` (`MAX_INSERT_SIZE`). Read pairs classified as dangling end and self-cycle ligation were discarded. PCR duplicates (redundant pairs with both reads at the same positions: about 13.6% of the initial material) were also filtered out.

3.4.4 Interaction matrix generation

Valid pairs were used to generate interaction matrices at various resolutions depending on the bins size. Most of the subsequent analyses were performed at the 500 Kb resolution apart from few exceptions (TAD detection for instance was performed at the 50 Kb resolution). A total of 6 interaction matrices were obtained per resolution ($n = 3$ (replicates) $\times$ 2 (groups)). Additionally, merged interaction matrices were computed by summing the interaction values of the 3 matrices for each group. Considering the high number of unassembled scaffolds in the pig genome Sscrofa11.1 version and given the fact that samples from both genders were collected, we focused our analysis on the 18 assembled autosomes to avoid potential effects of the sexual chromosomes on the results. Interaction matrices were displayed using Juicebox (Durand et al., 2016) and **HiTC** R / Bioconductor package v1.18.1 (Servant et al., 2012).

3.4.5 Within-matrix normalization

Interaction matrices were normalized per chromosome using the non-parametric iterative correction and eigenvector decomposition (ICE) method (Imakaev et al., 2012) as implemented in HiC-Pro (Servant et al., 2015). This method assumes an equal exposure to contacts for each region with all the others. 2% of bins showing the lowest counts were filtered out by fixing the following parameter: `FILTER_LOW_COUNT_PERC = 0.02`. The maximum number of iterations was also fixed to 100 (`MAX_ITER = 100`). Normalized matrices were used for A/B compartment finding but not for the differential analysis for which raw data were used together with a between-matrix normalization. To find TADs, raw matrices were used because matrix balancing was integrated within the TAD calling software.

3.4.6 Replicability

Replicability between interaction matrices was assessed using the replicability index of Yang et al. (2017) as implemented in the R / Bioconductor package **hicrep**. Intra-chromosome interaction matrices ($n = 3$ (replicates) $\times$ 2 (groups) $\times$ 18 (autosomes) $\times$ 2 resolutions) were extracted at 200 and 500 Kb resolutions. Low count interactions (total count over the 6 replicates < 30) were filtered out of the analysis to avoid artificially inflating the replicability index. For every chromosome and for the two resolutions, pairwise replicability indices were computed between any pair of intra-chromosome interaction matrices (15 indices $\times$ 18 (autosomes) were thus computed). These indices were averaged over the 18 autosomes to obtain a global replicability index for any pair of interaction matrices (15 averaged indices). Most of the statistical analyses were then performed using R (version 3.3.3, R Core Team (2017)).

3.4.7 Resolution computation

To define the maximal resolution that could be reached for a given interaction matrix, we applied the original definition proposed by Lieberman-Aiden et al. (2009): a given resolution (bin size) can be claimed if at that resolution 80% of the bins or more contain at least 1,000 interactions. The proportion of bins with a cumulated number of valid interactions higher than 1,000 was therefore computed for different resolutions (from 100 to 5 Kb) for each individual (sample) and merged (group) matrix.

3.4.8 TADs calling and comparison

TADs were predicted per chromosome from raw interaction matrices ($n = 3$ (replicates) $\times$ 2 (groups) $\times$ 18 (autosomes)) at 50 Kb resolution with the Arrowhead method of the Juicer tool v1.5.3, using the `-k KR` parameter to ensure matrix balancing normalization. TAD finding was performed on individual matrices of each replicate separately (to assess group replicability) and on the merged matrices ($n = 2$ (groups) $\times$ 18 (autosomes)) to obtain a set of TADs for each group (90/110 days of gestation). To identify TADs that are consistently predicted from different replicates and group-specific TADs, we performed pairwise comparisons of TAD sets from different replicates using bedtools (v2.26.0). A mutual overlap of 90% similarity was required with the parameters `-f 0.9 -r`.

Insulation capacity of TAD boundaries was computed as previously described (Foissac et al., 2019) using the local interaction score. In brief, considering all valid interactions around the same TAD boundary (*i.e.*, both reads being not further than 500 Kb from the boundary) the interaction score corresponds to the proportion of valid interactions across the boundary. Since such interactions should not often occur across "strong" TAD boundaries, the resulting interaction scores are expected to be low. Symmetrically, "weak" or "permissive" boundaries are expectedly assigned relatively higher interaction scores. Raw number of total and cross-boundary interactions were computed around each TAD for each of the 6 samples and further normalized using a cyclic loess approach (Ballman et al., 2004) implemented in the normOffsets function of the R / Bioconductor package **csaw** (Lun and Smyth, 2015) to correct trend biases in pairwise MA plots.

3.4.9 CTCF prediction

The position specific frequency matrix corresponding to the CTCF-binding motif was recovered from the JASPAR Transcription Factor Binding Sites (TFBS) catalogue (`http://jaspar.genereg.net`, Mathelier et al. (2016)). CTCF genomic occurrences were predicted by running FIMO v.4.11.1 Grant et al. (2011) with the JASPAR CTCF frequency matrix on the Sscrofa11.1 genome. Then, the average density of CTCF predicted motifs with respect to TAD positions was obtained using bedtools v2.26.0 map and coverage functions Quinlan (2014).

3.4.10 A/B compartments detection

A and B compartments were obtained using the PCA approach described in Lieberman-Aiden et al. (2009), as implemented in the R / Bioconductor package **HiTC** (Servant et al., 2012). A/B compartment identification was performed on intra-chromosome interaction matrices at 500 Kb resolution on individual interaction matrices ($n = 3$ (replicates) $\times$ 2 (groups) $\times$ 18 (autosomes)) and on the merged interaction matrix ($n = 18$ autosomes). Boundaries between A and B compartments were identified according to the sign of the first PC (eigenvector). Since PCA was performed on each chromosome separately, the average counts on the diagonal of the normalized matrix were used to identify which PC sign ($+/-$) should be assigned to A and B compartments for each chromosome (A compartments corresponded to the largest average counts in all chromosomes). This allowed to obtain a homogeneous assignment across chromosomes without using any annotation-based information like gene density or gene expression metrics. These metrics could therefore be used in subsequent QC validations to assess the relevance of the compartmentalization. In addition, some bins were not assigned to any compartment (due to a lack of data) in some samples and were not considered in subsequent integrative analyses. To test if the robustness of the compartment detection was significant across replicates (83.3% of agreement across the 6 samples) a permutation test was performed: A and B labels were shuffled across all genomic bins within each sample and the proportion of bins with the same label across the 6 samples was computed. None of the 1,000 permutations led to a percentage as high as 83.3% (p-value $< 10e^{-3}$). In addition, for quality control, A/B compartments were also obtained by using the eigenvalue method of the Juicer tool (Durand et al., 2016), which lead to similar results.

The difference between the number of compartments in the two groups was assessed with a Poisson GLM: $\log(y_{ijk}) \sim \alpha\, c_{ijk} + \beta_k$, with y_{ijk} the number of compartments in chromosome j from sample i in group k, c_{ijk} the total number of valid interactions in chromosome j from sample i in group k, α its estimated effect on the number of compartments, and β_k the estimated effect of the group on the number of compartments, which was tested for being significantly different from 0 (test with $n = 2$ (groups) $\times$ 3 (samples) $\times$ 18 (chromosomes) observations). This model was fit only for the number of compartments and not for the number of TADs since the latter was clearly driven by the number of valid interactions mainly.

3.4.11 Detection of differential interactions

A differential analysis was performed to extract interactions that were significantly differentially connected between the two groups (90 and 110 days of gestation). This analysis was performed on raw count data from the 18 autosomes at the 500 Kb resolution (the differential analysis was thus performed with 2 groups and $n = 3$ replicates in each group). A method similar to the one described in Lun and Smyth (2015), with some adaptations, was used to perform this task. More precisely, a 3-step approach was used which consisted in:

1. A filtering step, in which low count interactions were removed from the dataset: this step is used to leverage the effect of multiple testing correction (and to improve the testing procedure power) by removing low count interactions that have a very low chance to be found differentially expressed. We chose to use a fixed threshold based on the total number of reads, across the 6 samples, associated to a given interaction ($\tau = 30$, which corresponded to a minimum of 5 reads per sample on average), to filter out irrelevant bin pairs from the differential analysis.
2. A normalization step (between interaction matrix normalization), to make the different matrices comparable. As stressed in Lun and Smyth (2015), contrary to RNA-seq data, a normalization based on the (potentially corrected) library size is not sufficient for Hi-C data. Indeed, the complexity of the protocol generally generates additional biases that result in trended differences between libraries (as visible on MA plots). To correct such biases, we used the method proposed in Ballman et al. (2004) and implemented in the R / Bioconductor package **csaw** (Lun and Smyth, 2016) that performs a non-linear normalization based on a fast cyclic loess algorithm. Gene and sample specific offsets were computed and incorporated in the Generalized Linear Model (GLM) described in the next step, to correct trended differences. The efficiency of the normalization was controlled using PCA and MA plots on pseudo counts ($\log_2$ transformed counts), before and after normalization.
3. A differential analysis step: this step was performed using a Generalized Linear Model (GLM) based on the Negative Binomial (NB) distribution with a group fixed effect (two-level factor: 90/110 days). The model was estimated with the implementation of the R / Bioconductor package **edgeR** (McCarthy et al., 2012; Robinson et al., 2010) and log ratio tests were used to assess the significativity of the group effect on each bin pair interaction. p-values were genome wide corrected using (Benjamini and Hochberg, 1995) procedure to control the False Discovery Rate (FDR).

3.4.12 Characterization of BODIs

As a single genomic bin can be involved in multiple Differential Interactions (DI) genome-wide with various logFC values, we looked for bins with a large prevalence of interactions of the same logFC sign, either mostly positive or mostly negative. A minimum ratio of 90% of DI with the same sign was required to identify "positive" or "negative" bins, possibly indicating regions that undergo a chromatin contraction or opening, respectively. Bins with a mixture of positive and negative DI were considered as undefined. Adjacent bins with the same sign (either positive, negative, or undefined) were merged into Blocks Of Differential Interactions (BODIs). This analysis was performed considering only intra-chromosomal DIs (in *cis*).

To assess the existence of an enrichment of large positive and negative BODIs given the relative proportions of positive and negative individual DIs, a permutation test was performed: at each permutation, logFC values were shuffled genome-wide across DIs. The same 10:1 threshold was applied to define prevalently positive and negative bins and adjacent bins of the same type were merged to identify "expected BODIs" under the null hypothesis (no specific trend of positive/negative bins to cluster consecutively). The resulting size distributions of positive, negative and undefined BODIs were compared with that of observed

BODIs, and the p-value was computed, as the number of times expected BODIs were at least as frequent as the observed ones across 100 permutations for a given size and type.

The comparison of BODIs with A/B compartments was done by computing the proportion of the positive, negative and undefined BODIs that overlapped A or B compartments in terms of genomic space. The resulting block composition was therefore obtained using the bedtools coverage function on BODIs of each size and compartments of each type. As most of the compartmentalization is stable across samples, the A/B compartments obtained on the merged general matrix was used. Since A and B compartments cover roughly the same genomic space in total, no large difference should be observed between the A and B composition of positive and negative BODIs. Significance was assessed using Fisher's exact test between the compartment type (A/B) and the BODI types (positive/negative).

3.5 Gene expression integrative analysis

3.5.1 Expression data

Expression data were obtained from a previous transcriptome study of skeletal muscle in pig during development using microarrays (Voillet et al., 2014). The dataset consists of 44,368 probe measurements for 17 samples (LW animals) at two different gestational stages: 8 samples at 90 days and 9 samples at 110 days. A precise description of the experimental design and data collection can be found in Voillet et al. (2014). Normalized expression data ($\log_2$ transformed) and sample information are available in NCBI (GEO accession number GSE56301). $\log_2$ transformed expressions and log fold change (logFC) of these expression values at 90 vs. 110 days (reference time point: 90 days) were used in our integrative analyses. Since the microarray was originally designed on a former version of the pig genome, the probe sequences were mapped to the more recent Sscrofa11.1 assembly version by using BLAT (v.35x1) with the parameters `-minIdentity=95 -mask=lower`, in order to anchor expression data on reliable genomic positions. Stringent filtering steps were applied to keep only high quality hits. Alignments were obtained and processed by keeping only unique best hits with a minimum score of 30. When several "blocks" were found in the alignment of a given probe -across two exons for instance, the longest block (with a minimum length of 20) was kept. The 42,885 resulting probes were then annotated depending on their mapping position relatively to the annotated genes of the Ensembl v98 annotation. Probes that overlapped an annotated exon from the Ensembl annotation were assigned the corresponding gene ID. A total of 27,641 exonic probes could be unambiguously assigned to a gene, targeting 11,296 genes with an average of 2.5 probes per gene. Gene expression values were obtained by averaging the values of the corresponding probes.

3.5.2 Density and expression level of genes in A/B compartments

To compare the gene density in A vs. B compartments, a gene density value was first computed for each compartment by dividing the number of distinct gene IDs included in the compartment (using bedtools map) by the size of the compartment. Resulting gene density distributions were then compared between A and B compartments. Normality of the gene density was tested using Shapiro-Wilk normality test and rejected for all types of compartments in both groups (p-values $< 2.2e^{-16}$ overall, for $n = 349$ and 322 A and B compartments respectively). Wilcoxon tests were then used to assess the significance of the difference in gene density in A vs B compartments.

To compare the average gene expression in A vs. B compartment, we computed for each compartment the mean expression value of its genes using bedtools map separately for the two gestational ages. Normality of the average gene expression was tested using Shapiro-Wilk normality tests and rejected for both A and B compartments (p-values $= 2.58e^{-5}$ and $1.08e^{-3}$ for $n = 344$ and 292 A and B compartments with at

least one expressed gene, respectively). Wilcoxon tests were then used to assess the significance of the difference in gene expression in A vs B compartments.

To investigate the dynamic of expression in compartment-switching regions, we considered the logFC expression values of the genes and split them into compartment-switching categories using bedtools: no switch, A to B, B to A. Normality of the logFC expression values was tested using Shapiro-Wilk normality tests for genes in all types of compartments except for compartments with no switch ($n = 7,511$ genes in these compartments, above the applicability condition of the test) and rejected for both types of compartments (p-values $= 1.2e^{-3}$ and $4.6e^{-6}$, for $n = 60$ and 174 genes in compartments switching from A to B and from B to A, respectively). Wilcoxon tests were then used to assess the significance of the difference in logFC expression values in each compartment type.

REFERENCES

Ballman, K. V., Grill, D. E., Oberg, A. L., and Therneau, T. M. (2004). Faster cyclic loess: normalizing RNA arrays via linear models. *Bioinformatics* 20, 2778–2786. doi:10.1093/bioinformatics/bth327

Belton, J.-M., McCord, R. P., Gibcus, J. H., Naumova, N., Zhan, Y., and Dekker, J. (2012). Hi-C: a comprehensive technique to capture the conformation of genomes. *Methods* 58, 268–276. doi:10.1016/j.ymeth.2012.05.001

Benjamini, Y. and Hochberg, Y. (1995). Controlling the false discovery rate: a practical and powerful approach to multiple testing. *Journal of the Royal Statistical Society Series B* 57, 289–300

Durand, N. C., Shamim, M. S., Machol, I., Rao, S. S., Huntley, M. H., Lander, E. S., et al. (2016). Juicer provides a one-click system for analyzing loop-resolution Hi-C experiments. *Cell Systems* 3, 95–98. doi:10.1016/j.cels.2016.07.002

Foissac, S., Djebali, S., Munyard, K., Vialaneix, N., Rau, A., Muret, K., et al. (2019). Multi-species annotation of transcriptome and chromatin structure in domesticated animals. *BMC Biology* 17, 108. doi:10.1186/s12915-019-0726-5

Grant, C. E., Bailey, T. L., and Noble, W. S. (2011). FIMO: scanning for occurrences of a given motif. *Bioinformatics* 27, 1017–1018. doi:10.1093/bioinformatics/btr064

Iannuccelli, E., Mompart, F., Gellin, J., Lahbib-Mansais, Y., Yerle, M., and Boudier, T. (2010). NEMO: a tool for analyzing gene and chromosome territory distributions from 3D-FISH experiments. *Bioinformatics* 26, 696–697. doi:10.1093/bioinformatics/btq013

Imakaev, M., Fudenberg, G., McCord, R. P., Naumova, N., Goloborodko, A., Lajoie, B. R., et al. (2012). Iterative correction of Hi-C data reveals hallmarks of chromosome organization. *Nature Methods* 9, 999–1003. doi:10.1038/nmeth.2148

Lahbib-Mansais, Y., Barasc, H., Marti-Marimon, M., Mompart, F., Iannuccelli, E., Robelin, D., et al. (2016). Expressed alleles of imprinted IGF2, DLK1 and MEG3 colocalize in 3D-preserved nuclei of porcine fetal cells. *BMC Cell Biology* 17, 1–15. doi:10.1186/s12860-016-0113-9

Langmead, B. and Salzberg, S. L. (2012). Fast gapped-read alignment with Bowtie 2. *Nature Methods* 9, 357–359. doi:10.1038/nmeth.1923

Lieberman-Aiden, E., Van Berkum, N. L., Williams, L., Imakaev, M., Ragoczy, T., Telling, A., et al. (2009). Comprehensive mapping of long-range interactions reveals folding principles of the human genome. *Science* 326, 289–293. doi:10.1126/science.1181369

Lun, A. T. and Smyth, G. K. (2015). diffHic: a Bioconductor package to detect differential genomic interactions in Hi-C data. *BMC Bioinformatics* 16, 258. doi:10.1186/s12859-015-0683-0

Lun, A. T. and Smyth, G. K. (2016). csaw: a Bioconductor package for differential binding analysis of ChIP-seq data using sliding windows. *Nucleic Acids Research* 44, e45. doi:10.1093/nar/gkv1191

Marti-Marimon, M., Vialaneix, N., Voillet, V., Yerle-Bouissou, M., Lahbib-Mansais, Y., and Liaubet, L. (2018). A new approach of gene co-expression network inference reveals significant biological processes involved in porcine muscle development in late gestation. *Scientific Report* 8, 10150. doi:10.1038/s41598-018-28173-8

Mathelier, A., Fornes, O., Arenillas, D. J., Chen, C.-y., Denay, G., Lee, J., et al. (2016). JASPAR 2016: a major expansion and update of the open-access database of transcription factor binding profiles. *Nucleic Acids Research* 44, D110–D115. doi:10.1093/nar/gkv1176

McCarthy, D. J., Chen, Y., and Smyth, G. K. (2012). Differential expression analysis of multifactor RNA-Seq experiments with respect to biological variation. *Nucleic Acids Research* 40, 4288–4297. doi:10.1093/nar/gks042

Mompart, F., Robelin, D., Delcros, C., and Yerle-Bouissou, M. (2013). 3d organization of telomeres in porcine neutrophils and analysis of LPS-activation effect. *BMC Cell Biology* 14, 30. doi:10.1186/1471-2121-14-30

Quinlan, A. R. (2014). BEDTools: the Swiss-army tool for genome feature analysis. *Current Protocols in Bioinformatics* 47, 11.12.1–34. doi:10.1002/0471250953.bi1112s47

R Core Team (2017). *R: A Language and Environment for Statistical Computing*. R Foundation for Statistical Computing, Vienna, Austria

Robinson, M. D., McCarthy, D. J., and Smyth, G. K. (2010). edgeR: a Bioconductor package for differential expression analysis of digital gene expression data. *Bioinformatics* 26, 139–140. doi:10.1093/bioinformatics/btp616

Servant, N., Lajoie, B. R., Nora, E. P., Giorgetti, L., Chen, C.-J., Heard, E., et al. (2012). HiTC: exploration of high-throughput 'C' experiments. *Bioinformatics* 28, 2843–2844. doi:10.1093/bioinformatics/bts521

Servant, N., Varoquaux, N., Lajoie, B. R., Viara, E., Chen, C.-J., Vert, J.-P., et al. (2015). HiC-Pro: an optimized and flexible pipeline for Hi-C data processing. *Genome Biology* 16, 259. doi:10.1186/s13059-015-0831-x

Voillet, V., SanCristobal, M., Lippi, Y., Martin, P. G., Iannuccelli, N., Lascor, C., et al. (2014). Muscle transcriptomic investigation of late fetal development identifies candidate genes for piglet maturity. *BMC Genomics* 15, 797. doi:10.1186/1471-2164-15-797

Wickham, H., Averick, M., Bryan, J., Chang, W., D'Agostino McGowan, L., François, R., et al. (2019). Welcome to the tidyverse. *Journal of Open Source Software* 4, 1686. doi:10.21105/joss.01686

Yang, T., Zhang, F., Yardimci, G. G., Song, F., Hardison, R. C., Noble, W. S., et al. (2017). HiCRep: assessing the reproducibility of Hi-C data using a stratum-adjusted correlation coefficient. *Genome Research* 27, 1939–1949. doi:10.1101/gr.220640.117
